# Hypochlorous acid derived from microglial myeloperoxidase could mediate high-mobility group box 1 release from neurons to amplify brain damage in cerebral ischemia–reperfusion injury

**DOI:** 10.1186/s12974-023-02991-8

**Published:** 2024-03-21

**Authors:** Shuang Chen, Jingrui Pan, Zhe Gong, Meiling Wu, Xiaoni Zhang, Hansen Chen, Dan Yang, Suhua Qi, Ying Peng, Jiangang Shen

**Affiliations:** 1 School of Chinese Medicine, Li Ka Shing Faculty of Medicine, University of Hong Kong, Hong Kong, Hong Kong SAR China; 2 Department of Neurology, Sun Yat-Sen Memorial Hospital, Sun Yat-Sen University, Guangzhou, 510120 China; 3 Department of Chemistry, University of Hong Kong, Hong Kong, Hong Kong SAR China; 4 Medical and Technology School, Xuzhou Key Laboratory of Laboratory Diagnostics, Xuzhou Medical University, Xuzhou, China; 5 Guangdong Provincial Key Laboratory of Malignant Tumor Epigenetics and Gene Regulation, Sun Yat-Sen Memorial Hospital, Sun Yat-Sen University, Guangzhou, China

**Keywords:** Hypochlorous acid, Myeloperoxidase, High-mobility group box 1, Stroke, Microglia

## Abstract

Myeloperoxidase (MPO) plays critical role in the pathology of cerebral ischemia–reperfusion (I/R) injury via producing hypochlorous acid (HOCl) and inducing oxidative modification of proteins. High-mobility group box 1 (HMGB1) oxidation, particularly disulfide HMGB1 formation, facilitates the secretion and release of HMGB1 and activates neuroinflammation, aggravating cerebral I/R injury. However, the cellular sources of MPO/HOCl in ischemic brain injury are unclear yet. Whether HOCl could promote HMGB1 secretion and release remains unknown. In the present study, we investigated the roles of microglia-derived MPO/HOCl in mediating HMGB1 translocation and secretion, and aggravating the brain damage and blood-brain barrier (BBB) disruption in cerebral I/R injury. In vitro, under the co-culture conditions with microglia BV cells but not the single culture conditions, oxygen–glucose deprivation/reoxygenation (OGD/R) significantly increased MPO/HOCl expression in PC12 cells. After the cells were exposed to OGD/R, MPO-containing exosomes derived from BV2 cells were released and transferred to PC12 cells, increasing MPO/HOCl in the PC12 cells. The HOCl promoted disulfide HMGB1 translocation and secretion and aggravated OGD/R-induced apoptosis. In vivo, SD rats were subjected to 2 h of middle cerebral artery occlusion (MCAO) plus different periods of reperfusion. Increased MPO/HOCl production was observed at the reperfusion stage, accomplished with enlarged infarct volume, aggravated BBB disruption and neurological dysfunctions. Treatment of MPO inhibitor 4-aminobenzoic acid hydrazide (4-ABAH) and HOCl scavenger taurine reversed those changes. HOCl was colocalized with cytoplasm transferred HMGB1, which was blocked by taurine in rat I/R-injured brain. We finally performed a clinical investigation and found that plasma HOCl concentration was positively correlated with infarct volume and neurological deficit scores in ischemic stroke patients. Taken together, we conclude that ischemia/hypoxia could activate microglia to release MPO-containing exosomes that transfer MPO to adjacent cells for HOCl production; Subsequently, the production of HOCl could mediate the translocation and secretion of disulfide HMGB1 that aggravates cerebral I/R injury. Furthermore, plasma HOCl level could be a novel biomarker for indexing brain damage in ischemic stroke patients.

**Supplementary Information:**

The online version contains supplementary material available at 10.1186/s12974-023-02991-8.

## Introduction

Neurovascular units (NVUs) are structural and functional multicellular modules composed of neurons, perivascular astrocytes, microglia, pericytes, extracellular matrix and endothelial cells. The NVUs provide a coordinated neurovascular coupling to maintain the selectivity of the blood–brain barrier (BBB) for the homeostasis of the central nervous system (CNS). The crosstalk and communications of the cells within NVUs and circulating inflammatory cells amplify brain damage and aggravate the BBB disruption and neurological deficits in stroke and traumatic brain injury (TBI) [1, 2]. Microglia are essential immune cells resident in CNS, act as the primary responders of the defense system and perform various functions based on dynamic communication with neurons and other neighboring cells. At the onset of stroke, the communication between neurons and microglia promotes the production of pro-inflammatory mediators that exacerbate neuronal damage in this vicious self-amplifying cycle [3, 4].

Extracellular vesicles (EVs) could affect the neighboring cells by secreting cellular signaling and factors. Microglia-derived exosomes mediate inflammatory responses by releasing pro-inflammatory mediators such as TNF-alpha, IL-1β, and miR-155 in TBI [5]. Circulating pro-inflammatory exosomes worsen stroke outcomes [6]. Exosomes released from microglia affect neuronal survival in ischemic stroke [7]. Myeloperoxidase (MPO) is highly expressed in activated microglia [8, 9] and widely distributed in ischemic brain tissues, aggravating ischemic brain injury [10]. The MPO-mediated oxidative stress and inflammation responses become promising therapeutic targets for ischemic stroke [11]. Exosomal MPO exerts as a biomarker of deep venous thrombosis [12]. However, whether microglial exosome-mediated MPO release affects the functions of adjacent neuronal cells in ischemic brain injury remains unknown.

Hypochlorous acid (HOCl), induced by the MPO–H_2_O_2_–Cl^−^ system, is the major cytotoxic factor contributing to MPO-mediated injury. With strong diffusible capacity, HOCl induces chlorinative stress and oxidizes lipids, proteins and DNA [13, 14] and activates the infiltrated neutrophils and monocytes [15–17]. HOCl exerts its cytotoxic properties in different experimental systems including both the in vitro cultured neuronal and endothelial cells [18, 19], and the in vivo rodent ischemic stroke models [8, 9, 20, 21]. As a potent oxidant, HOCl targets proteins with the thiol group and causes reversible or irreversible modifications [22–24]. For example, HOCl promotes the formation of two disulfide bonds of cysteines in Hsp33 and activates the molecular chaperone [25]. High-mobility group box 1 (HMGB1) is a representative inflammatory factor participating in ischemic brain injury. Serum HMGB1 level was significantly increased in ischemic stroke patients [26–28]. The translocation and release of HMGB1 trigger cytokine release and recruit leucocytes into ischemic penumbra [29, 30], amplifying plural inflammatory responses, and increasing infarct sizes and the BBB permeability [28, 31, 32]. Importantly, the formation of disulfide bond is a prerequisite for HMGB1 to cytoplasmic transportation and extracellular secretion [33, 34]. The secretion of disulfide HMGB1 is responsible for activating TLR4 signaling and initiating inflammation [35, 36]. The disulfide HMGB1 activates neuroinflammation and causes the BBB disruption in stroke and TBI [37]. Whether HOCl induces disulfide HMGB1 formation in ischemic brain injury remains unknown.

In the present study, we tested the hypothesis that the microglial MPO-containing exosomes increase adjacent neuronal HOCl production and mediate disulfide HMGB1 translocation, subsequently aggravating the ischemic brain injury and neurological deficits in cerebral ischemia–reperfusion (I/R) injury. The study highlights that microglial cells drive neuronal damage in the NVUs through releasing MPO-containing exosomes during ischemic brain injury.

## Results

### Microglia-derived MPO/HOCl production triggers neuronal apoptosis in a co-cultured system under oxygen–glucose deprivation and reoxygenation (OGD/R) in vitro

**Fig. 1:**
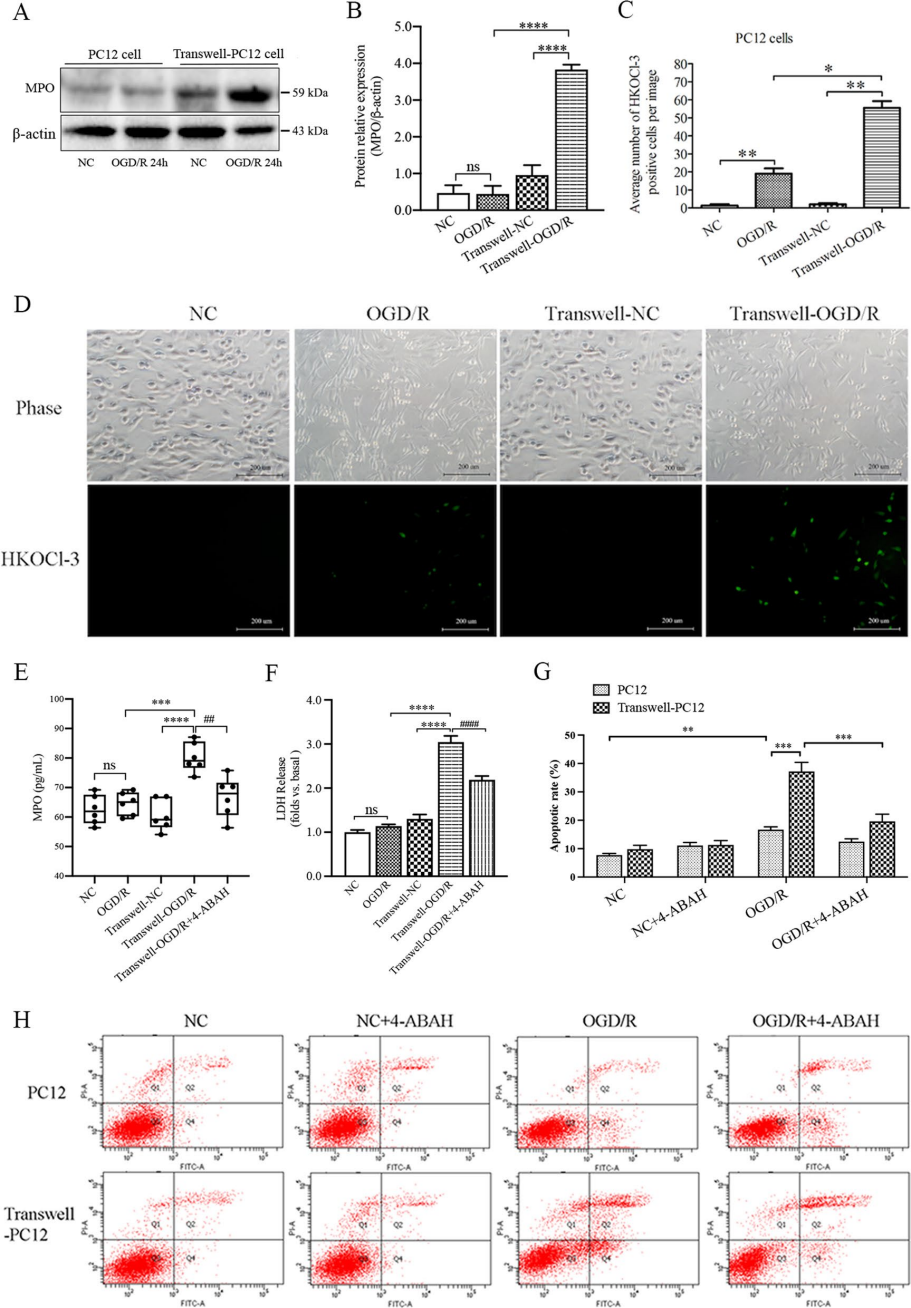
MPO expression and HOCl production in PC12 cells under monoculture or co-cultured with microglial BV2 cells after exposure to normoxic conditions (NC) or OGD/R in vitro. PC12 cells were mono-cultured or transwell co-cultured with BV2 cells and then exposed to 4 h OGD (glucose-free medium with 1% O2/5% CO2) or normal conditions (NC, normal glucose medium with 21% O2/5% CO2), and then the cells were cultured with fresh normal glucose medium with 21% O2/5% CO2 and incubated for 24 h. The cells were treated with 4-ABAH (50 µM) at the onset of reoxygenation. **A**, **B** Western blot analysis for MPO expression in PC12 cells under monoculture or co-culture with BV2 cells conditions. **C**, **D** HKOCl-3 staining HOCl production from PC12 cells in single cultured plates or co-cultured transwells with BV2 cells, bar = 200 μm. **E** Quantification of MPO level in the supernatant by ELISA. **F** Necrotic cell death was detected by LDH release from PC12 cells. **G**, **H** Annexin V-FITC/PI staining flow cytometry assay for apoptotic cell death. Transwell-OGD/R: BV2 and PC12 co-cultured cells were exposed to 4 h OGD with 24 h of reoxygenation. *p < 0.05, **p < 0.01, ***p < 0.001, ****p < 0.0001, ##*p* < 0.01, ####*p* < 0.0001. All data are presented as mean ± SEM. (Statistical methods: **B**, **C**, **E**, **F** one-way ANOVA followed by Tukey’s multiple comparisons test. **G** Two-way ANOVA followed by Bonferroni multiple comparisons test.)

We first investigated the effects of microglia-derived MPO/HOCl production on adjacent neuronal cells in the transwell co-culture conditions after exposure to OGD/R treatment in vitro. The PC12 cells were independently cultured or co-cultured with microglial BV2 cells, then exposed to OGD for 4 h, followed by reoxygenation for 24 h with fresh medium to mimic I/R. We used our newly developed highly sensitive and specific fluorescent probe HKOCl-3 to label HOCl production [38]. After exposed to OGD/R, under single culture conditions, the PC12 cells had no significant changes in the expression of MPO (Fig. 1A, B) with slightly increased HOCl production (Fig. 1C, D). When co-cultured with BV2 cells, after OGD/R-24 h, the PC12 cells had significantly increased MPO expression and HOCl production (Fig. 1A–D). The expression of MPO in transwell-PC12 cells was increased at all detected timepoints (6, 12, 24 h) of reoxygenation (Additional file 1: Fig. S1). Meanwhile, the release of LDH (lactate dehydrogenase) and apoptotic cell death were significantly increased in the transwell-cultured PC12 cells (Fig. 1F–H). Treatment with 4-ABAH, an MPO inhibitor, significantly decreased the MPO level in the supernatant and reduced the LDH release and apoptotic cell death (Fig. 1E–H). Those results suggest that microglial cells might contribute to the MPO-derived HOCl production in the transwell-cultured PC12 cells under OGD/R conditions.

**Fig. 2:**
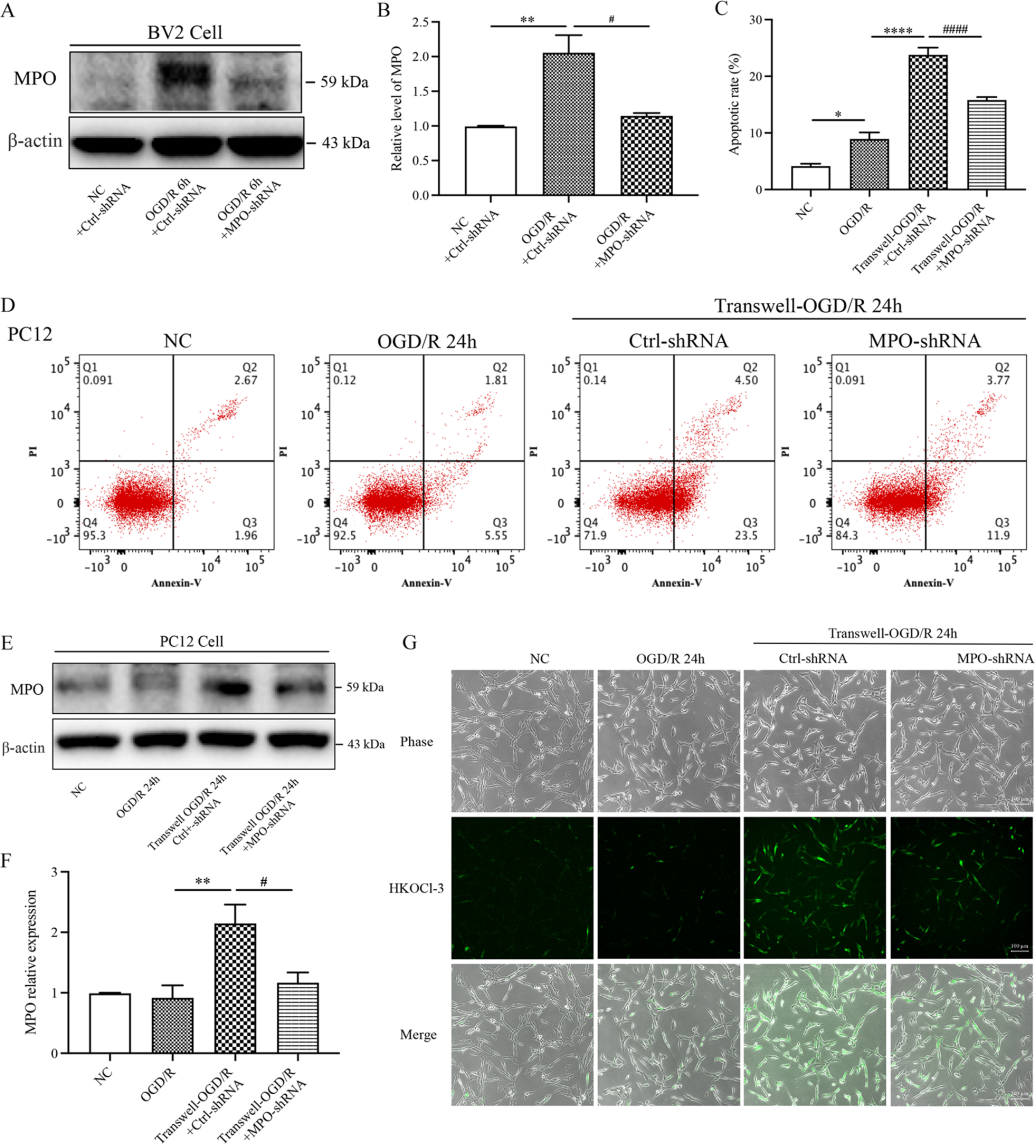
Genetic knockdown MPO expression in BV2 cells decreased HOCl production and attenuated apoptosis in PC12 cells under co-cultured conditions with OGD/R exposure. In the transwell system, PC12 cells were co-cultured with BV2 cells transfected with non-targeted shRNA (Ctrl-shRNA) or MPO-targeted shRNA (MPO-shRNA). Transwell-OGD/R: BV2 and PC12 co-cultured cells were exposed to 4 h OGD with 24 h of reoxygenation. **A**, **B** Western blot analysis for MPO expression in BV2 cells transfected Ctrl-shRNA or MPO-shRNA after OGD/R stimulation. **C**, **D** Annexin V-FITC/PI staining flow cytometry assay for apoptotic cell death. **E**, **F** Western blot analysis for MPO expression in PC12 cells among the groups. **G** HKOCl-3 staining HOCl production from PC12 cells among the groups, bar = 200 μm. *p < 0.05, **p < 0.01, ****p < 0.0001, #*p* < 0.05, ####*p* < 0.0001. All data are presented as mean ± SEM. (Statistical methods: **B**, **C**, **F** one-way ANOVA followed by Tukey’s multiple comparisons test.)

We then further tested the roles of MPO/HOCl derived from BV2 cells in mediating PC12 cell damages under co-cultured conditions with OGD/R exposure. The expression of MPO in BV2 cells was knocked down by transfected MPO-shRNA. The MPO-shRNA transfection effectively abolished the OGD/R-induced MPO expression in the BV2 cells (Fig. 2A, B). Then, the transfected BV2 cells were co-cultured with PC12 cells. Under OGD/R conditions, the transwell co-cultured PC12 cells with the vehicle-transfected BV2 cells had significantly higher rates of apoptotic cell death than the single-cultured PC12 cells. When co-cultured with the MPO-shRNA BV2 cells, the PC12 cells significantly attenuated the apoptosis (Fig. 2C, D). These results strongly support that the presence of MPO in BV2 cells plays a pivotal role in inducing adjacent neural cell death under transwell co-cultured conditions after OGD/R exposure. Furthermore, we also detected the production of MPO and HOCl in the PC12 cells. Interestingly, the co-culture with the MPO-shRNA BV2 cells significantly decreased the expression of MPO protein (Fig. 2E, F) and the level of HOCl production in the PC12 cells (Fig. 2G). These results further support the notion that MPO-derived from BV2 cells increases HOCl production and aggravates adjacent neural cell death under co-cultured conditions with the OGD/R exposure.

### Activated microglial cells release MPO-containing exosomes, and increase HOCl production in adjacent neuronal cells, aggravating apoptotic cell death under OGD/R conditions in vitro

**Fig. 3:**
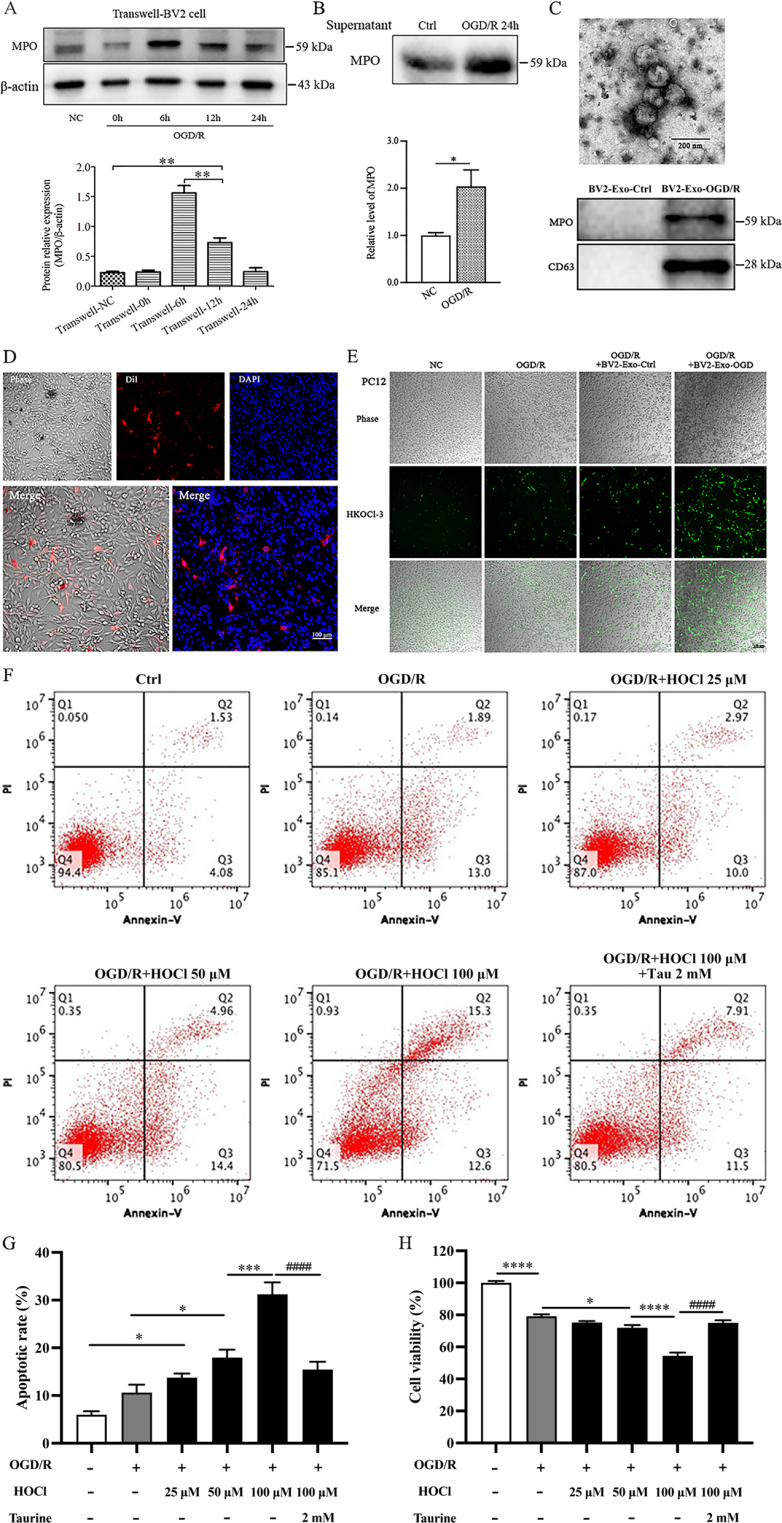
MPO-containing exosomes derived from microglial BV2 cells transfer into co-cultured PC12 cells, elevate HOCl level, and induce apoptosis in vitro. Exosomes were collected from the supernatant of BV2 cells after OGD/R-24 h exposure. PC12 cells were treated with HOCl (25, 50, 100 µM) in HBSS for 15 min after exposure to 4 h OGD and re-incubated in normoxic conditions (NC) for 24 h. Taurine (2 mM) was added at the onset of HOCl exposure. **A** MPO expression in BV2 cells after co-cultured with PC12 cells under NC or OGD/R conditions. **B** MPO expression in the supernatant of BV2 cells. supernatant of BV2 cells. **C** Upper panel, exosome image determined by TEM, bar = 200 nm; lower panel, expression of MPO and exosome marker CD63 in BV2-derived exosome. **D** Uptake of Dil-labeled exosomes by PC12 cells after cultured for 4 h. Bar = 100 μm. **E** HOCl production in PC12 cells treated with PBS, BV2-Exo-Ctrl or BV2-Exo-OGD/R under NC or OGD/R-24 h, bar = 100 μm. **F**, **G** Annexin V-FITC/PI staining flow cytometry for apoptotic cell death. **H** MTT assay for cell viability in PC12 cells. Transwell-NC: co-culture BV2 and PC12 cells under normal culture conditions; Transwell-0 h, 6 h, 12 h, 24 h: BV2 and PC12 co-cultured cells were exposed to 4 h OGD with 0 h, 6 h, 12 h, and 24 h of reoxygenation; BV2-Exo-Ctrl: exosomes released from NC-conditioned BV2 cells; BV2-Exo-OGD/R: exosomes released from OGD/R-stimulated BV2 cells. *p < 0.05, **p < 0.01, ***p < 0.001, ****p < 0.0001, ####*p* < 0.0001. All data are presented as mean ± SEM. (Statistical methods: **A**, **G**, **F** one-way ANOVA followed by Tukey’s multiple comparisons test; **B** two-tailed *t*-test.)

We then investigated the expression of MPO in the BV2 microglia cells co-cultured with PC-12 cells under OGD/R conditions. After the BV2 cells were exposed to OGD/R, the expression of MPO was temporarily increased at 6 h of reoxygenation (Transwell-6 h) but decreased at 12 h and 24 h of reoxygenation (Transwell-12 h, Transwell-24 h) (Fig. 3A). Interestingly, the level of MPO was increased in the supernatant from the transwell-cultured cells at OGD/R-24 h (Fig. 3B). Thus, we speculated that the potential release of MPO from the microglia cells as microglia could release exosomes to aggravate neuroinflammation [39]. As a result, we isolated exosomes from the supernatants of the BV2 cells under both NC and OGD/R conditions. The diameter of the exosome was around 100 nm (Fig. 3C, upper) and MPO was identified in the exosomes with exosomal marker protein CD63 (Fig. 3C, low). We then examined the effects of microglial exosome-mediated MPO release on HOCl production in the PC12 cells. The BV2-derived exosomes (BV2-Exo) were labeled with CM-Dil dye and incubated with PC12 cells for 4 h. The Dil-labeled exosomes were found in the cytoplasm, indicating the up-taken of exosomes by the PC12 cells (Fig. 3D). Interestingly, the exosomes released from OGD/R-stimulated BV2 cells (BV2-Exo-OGD/R) had a higher capacity to induce HOCl production in the PC12 cells than that from normal BV2 cells (BV2-Exo-Ctrl) (Fig. 3E). These results indicate that the activated microglial cells could release the MPO-containing exosomes to increase HOCl production in adjacent neuronal cells under OGD/R conditions. We next determined whether treatment of HOCl would aggravate apoptotic cell death in the PC12 cells under OGD/R conditions. As expected, HOCl dose-dependently increased apoptotic cell death and decreased cell viability. The cytotoxic effect was abolished by co-treatment of taurine, a HOCl scavenger (Fig. 3F–H). Those results suggest that microglia-derived MPO/HOCl production could mediate adjacent neuronal cell death under OGD/R conditions.

### HOCl induces disulfide HMGB1 formation and promotes its translocation and secretion in vitro

**Fig. 4:**
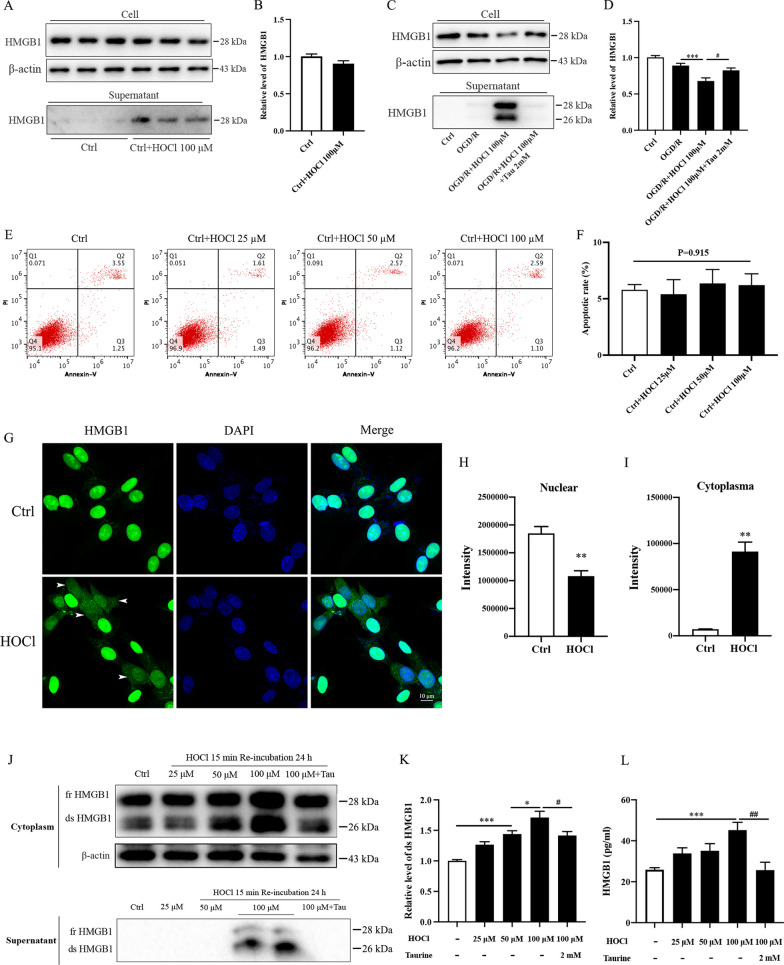
HOCl promotes translocation and secretion of disulfide HMGB1 from PC12 cells. The PC12 cells were treated with HOCl (25, 50, 100 µM, dissolved in HBSS) for 15 min after exposure to 4 h OGD or normoxic conditions (NC), and then incubated under NC for 24 h. Taurine (2 mM) was added at the onset of HOCl exposure. **A** Western blot revealing the expression of HMGB1 in PC12 cells and the supernatant at 24 h of re-incubation under NC conditions. **B** Quantitative analysis of HMGB1 in the cells. **C** Western blot revealing the expression of HMGB1 in PC12 cells and the supernatant after treatment of 4 h OGD plus 24 h reoxygenation. **D** Quantitative analysis of HMGB1 in the cells. **E**, **F** Annexin V-FITC/PI staining flow cytometry detection for apoptotic cell death by using apoptotic detection kit. **G** Immunofluorescent detection for HMGB1 location in the PC12 cells with or without HOCl treatment. **H** Quantitative analysis of HMGB1 level in nuclear with or without HOCl treatment. **I** Quantitative analysis of HMGB1 level in the cytoplasm with or without HOCl treatment. **J** Western blot showing the fully reduced HMGB1 (fr-HMGB1) and disulfide-HMGB1 (ds-HMGB1) in cytoplasm and supernatant of the PC12 cells after 4 h OGD plus 24 h reoxygenation. **K** Quantitative analysis of ds-HMGB1 in the cytoplasm. **L** ELISA detection of HMGB1 secretion in the supernatant of PC 12 cells after 4 h OGD plus 24 h reoxygenation. *p < 0.05, **p < 0.01, ***p < 0.001, #*p* < 0.05, ##*p* < 0.01. All data are presented as mean ± SEM. (Statistical methods: **B**, **H**, **I** two-tailed *t*-test; **D**, **K**, **L** one-way ANOVA followed by Tukey’s multiple comparisons test.)

HMGB1 is a representative inflammatory factor amplifying inflammatory responses in cerebral I/R injury [40]. Oxidative stress promotes the translocation and release of HMGB1 [41, 42]. We then tested whether HOCl could mediate HMGB1 translocation and secretion in the PC12 cells under OGD/R conditions. As expected, the release of HMGB1 from PC12 cells to the supernatants was confirmed after the cells were treated with HOCl under both NC and OGD/R conditions (Fig. 4A–D). HMGB1 could be translocated by active release or passive release based on the plasma membrane integrity. To test whether HOCl mediates active secretion of HMGB1, we treated the PC12 cells with HOCl (25, 50, and 100 μM) under normoxic conditions. No apoptotic cell death was induced by the treatment, as shown by Annexin V-FITC/PI assay (Fig. 4E, F). Immunofluorescent analysis revealed that HOCl directly induced the active nucleocytoplasmic translocation (marked with arrows) and secretion of HMGB1 in the PC12 cells (Fig. 4G–I). Similarly, HOCl also promoted the nucleocytoplasmic translocation of HMGB1 in primary neurons (Additional file 1: Fig. S2). Those results indicate that HOCl could mediate the active release of HMGB1 in both PC12 cells and primary cultured neurons.

The formation of disulfide bond between Cys23 and Cys45 is required to translocate HMGB1 from the nucleus into the cytoplasm requires [33]. Whether HOCl could induce disulfide HMGB1 and initiate the translocation and secretion of HMGB1 remains unclear. Thus, we investigated the level of disulfide HMGB1 in cytoplasm and supernatant. Western blot analysis showed that HOCl dose-dependently increased disulfide HMGB1 in cytoplasm and supernatant (Fig. 4J, K). ELISA revealed that HOCl treatment increased the HMGB1 level in the supernatant which was reversed by taurine (Fig. 4L). These results indicate that HOCl could promote the formation of disulfide HMGB1 and induce the active translocation and secretion of HMGB1.

### MPO/HOCl is increased in neurons, endothelial cells, and microglia of ischemic brains during cerebral I/R injury

**Fig. 5:**
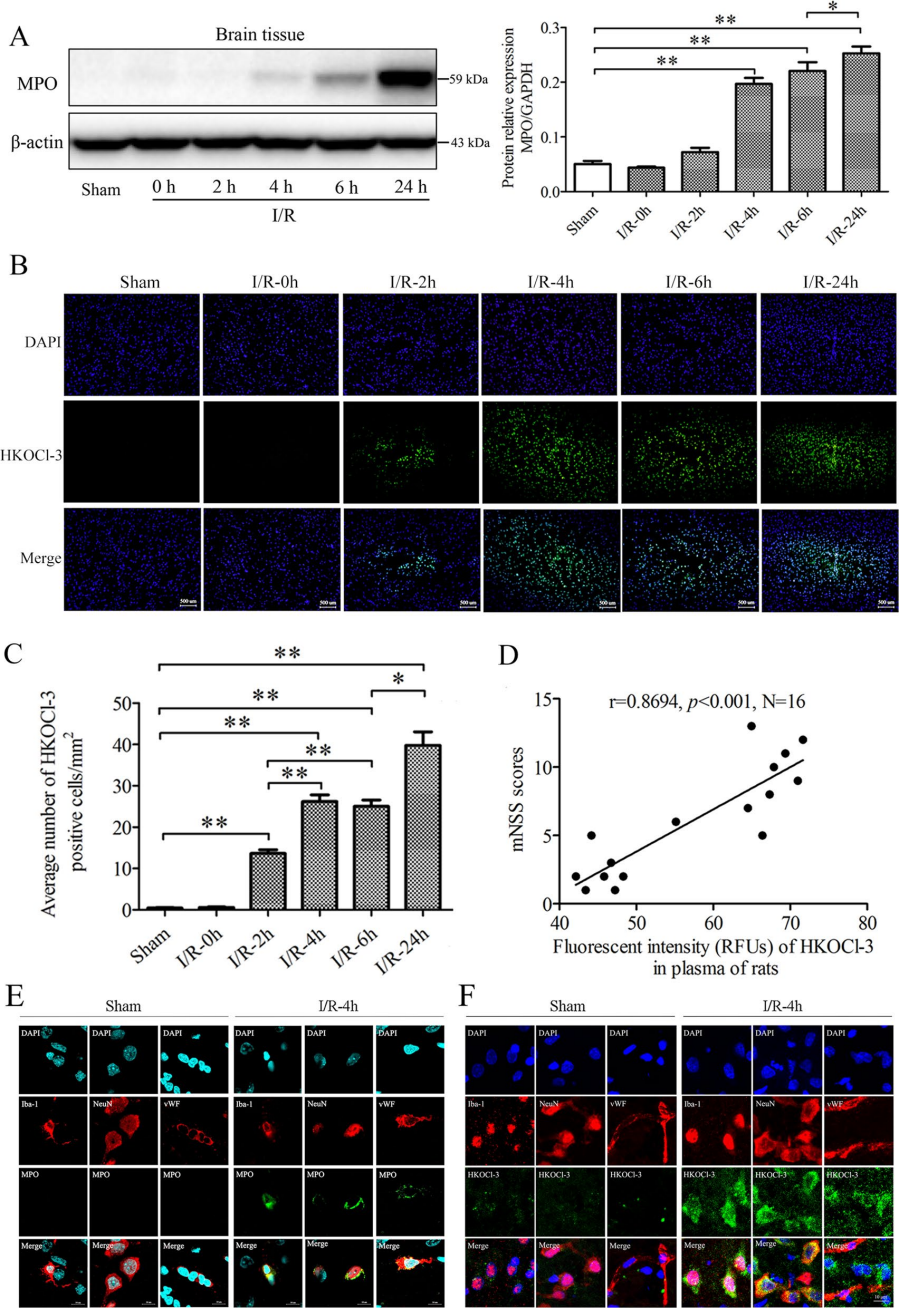
Dynamic changes and localization of MPO expression and HOCl production in ischemic brains during cerebral ischemia–reperfusion injury. Rats were subjected to 2 h of MCAO ischemia alone (I/R-0 h) or 2 h of MCAO ischemia plus 2, 4, 6, 24 h of reperfusion, termed as I/R-2 h, I/R-4 h, 1/R-6 h and I/R-24 h, respectively. MPO expression and HOCl production were detected by western blot analysis and HKOCl-3 staining fluorescence. **A** MPO expression in brain tissues; **B** HOCl level in rat brains. Bar = 500 μm. **C** Quantitative results of HOCl level in rat brains. **D** Pearson correlation coefficients for correlation between plasma HOCl level and mNSS in rat MCAO I/R injury. *N* = 16, *r* = 0.8694, *p* < 0.001. **E** Fluorescent imaging of MPO expression in microglia, neuron, and microvascular endothelial cells of the ischemic cortical area by co-stained with Iba-1, NeuN or vWF, respectively. Nucleus was stained with DAPI, bar = 10 μm. **F** HKOCl-3 staining fluorescent imaging for HOCl production in microglia, neuron, and microvascular endothelial cells co-stained with Iba-1, NeuN or vWF, respectively, nucleus was stained with DAPI, bar = 10 μm. *p < 0.05, **p < 0.01. All data are presented as mean ± SEM. (Statistical methods: **A**, **C** one-way ANOVA followed by Tukey’s multiple comparisons test; **D** Pearson correlation coefficient.)

We next investigated the dynamic changes and distributions of MPO/HOCl in a rat model of acute cerebral I/R injury in vivo. The rats were subjected to 2 h of MCAO cerebral ischemia followed by 0, 2, 4, 6 or 24 h of reperfusion, referring to I/R-0, I/R-2 h, I/R-4 h, I/R-6 h and I/R-24 h, respectively (Fig. 5). There was no significant difference in the MPO expression and HOCl production in the sham control and 2 h MCAO ischemia rats (I/R-0 h) (Fig. 5A). However, both MPO and HOCl in the ischemic cortex adjacent to subcortical regions were increased with reperfusion time (I/R-4 h, I/R-6 h, I/R-24 h) (Fig. 5A–C). The increased HOCl level was correlated with the increased neurobehavioral deficit scores in the cerebral I/R rats (Fig. 5D). We then identified the distributions of MPO/HOCl in the neurons, endothelial cells, microglia, and astrocytes by using NeuN, vWF, Iba-1 and GFAP, respectively. Interestingly, both MPO and HOCl were increased in the neurons, endothelial cells, and microglia of the post-ischemic brains (Fig. 5E, F). Neither MPO nor HOCl was detected in the astrocytes (Additional file 1: Fig. S3). These results indicate that the increased MPO/HOCl production could be found in neurons, endothelial cells, and microglia of the ischemic brains during cerebral I/R injury.

### MPO inhibitor 4-ABAH and HOCl scavenger taurine attenuate neurological deficits, infarct volume and BBB disruption in cerebral I/R injury

**Fig. 6:**
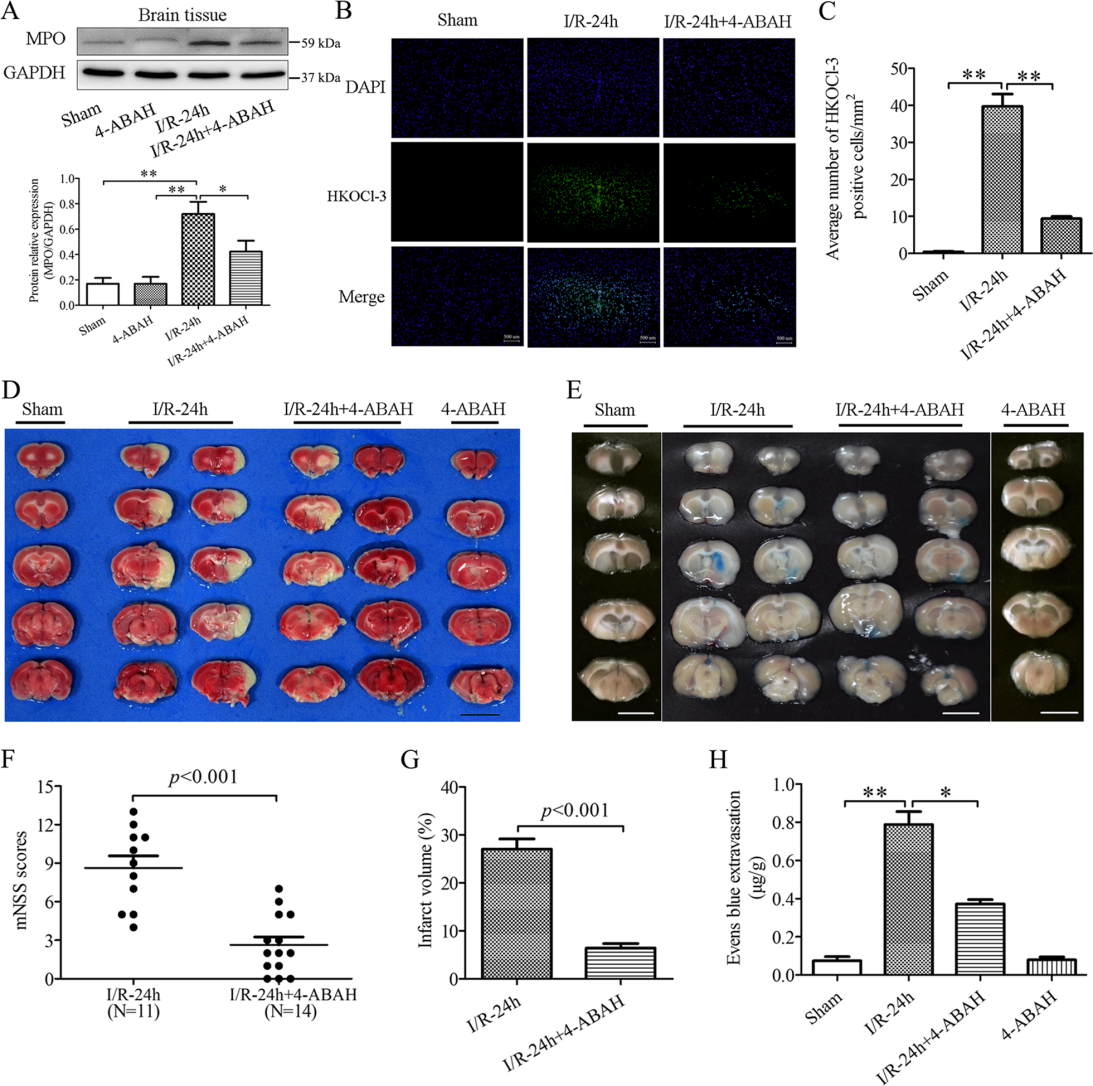
MPO inhibitor 4-ABAH reduced MPO/HOCl production, attenuated infarct volume and BBB disruption, and improved neurological functions in rat brains after MCAO ischemia–reperfusion (I/R). I/R rats were subjected to 2 h of MCAO ischemia plus 24 h of reperfusion. Sham control rats were subjected to similar operation without MCAO. MPO inhibitor 4-ABAH (20 mg/kg wt.) was intraperitoneally injected at the onset and 12 h of reperfusion, respectively (**A**) Western blot analysis for MPO expression. **B**, **C** HOCl level in I/R brains. **D**, **G** TTC staining and quantitative analysis showing infarct volumes in sham control and I-2 h/R-24 h rat brains with or without 4-ABAH treatment. **E**, **H** Evans blue leakage in rat brains of sham control and I-2 h/R-24 h with or without 4-ABAH treatment. **F** Neurological deficit scores (mNSS) in I-2/R-24 h rats with or without 4-ABAH treatment. *p < 0.05, **p < 0.01. All data are presented as mean ± SEM. (Statistical methods: **A**, **C**, **H** one-way ANOVA followed by Tukey’s multiple comparisons test; **F**, **G** two-tailed *t*-test.)

**Fig. 7:**
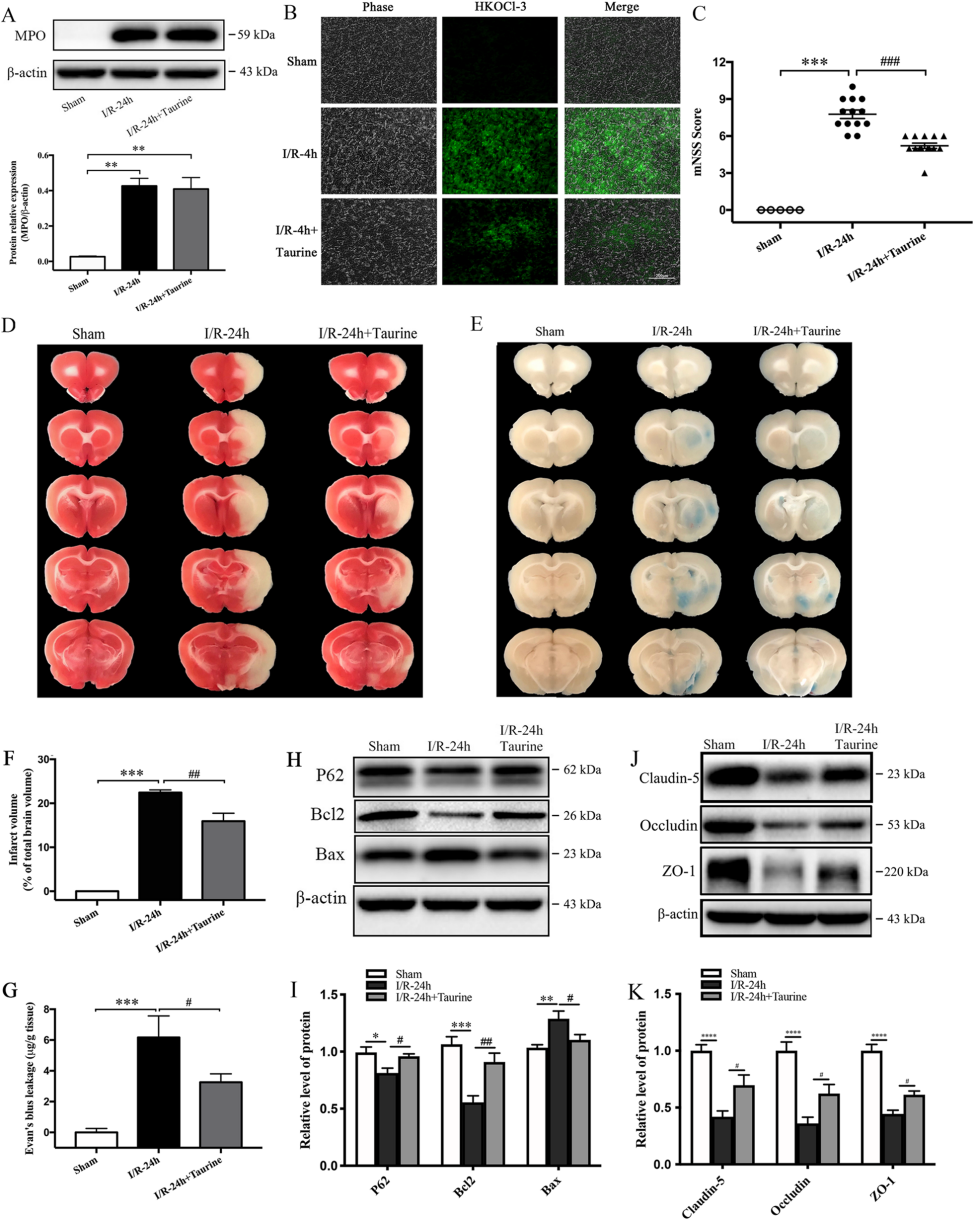
HOCl scavenger taurine reduced infarct volume, attenuated BBB disruption, and improved neurological function in rat brains after MCAO ischemia–reperfusion (I/R). I/R rats were subjected to 2 h of MCAO ischemia plus 24 h of reperfusion. Sham control rats were subjected to similar operation without MCAO. Taurine (Tau, 50 mg/kg) was intravenously administered at the onset of reperfusion. MPO expression and HOCl production were detected by western blot analysis and HKOCl-3 staining immunofluorescence, respectively. Infarct volumes were detected by TTC staining and quantitative analysis. **A** MPO expression in brain tissues. **B** HOCl production in the cortex of ischemic rat brains, bar = 200 μm. **C** Neurological deficit scores (mNSS). **D**, **F** Infarct volumes. **E**, **G** Evans blue leakage for the BBB permeability in I/R rat brains. **H**–**I** Western blot and quantitative analysis for P62, Bcl2 and Bax expression in I/R rat brains. **J**, **K** Western blot and quantitative analysis for tight junction proteins Claudin-5, Occludin and ZO-1 expression in I/R rat brains. *p < 0.05, **p < 0.01, ***p < 0.001, ****p < 0.0001 compared to Sham group. #*p* < 0.05, ##*p* < 0.01, ###*p* < 0.001, compared to I-2 h/R-24 h group. All data are presented as mean ± SEM. (Statistical methods: **A**, **I**, **K** one-way ANOVA followed by Tukey’s multiple comparisons test; **C**, **F**, **G** one-way ANOVA followed by Bonferroni multiple comparisons test.)

We then investigated the neuroprotective effects of 4-ABAH and taurine against cerebral I/R injury. As expected, 4-ABAH treatment downregulated the expression of MPO and decreased the HOCl production in the MCAO ischemic brain tissues (Fig. 6A–C). Meanwhile, the 4-ABAH treatment effectively reduced infarct sizes (Fig. 6D, G), protected the BBB integrity (Fig. 6E, H), and decreased the neurological deficit scores (Fig. 6F). We also investigated apoptotic cell death in the neurons, microglial cells, and brain endothelial cells by using co-immunostaining of the TUNEL with their respective markers, including NeuN, Iba-1, and CD31. The treatment of 4-ABAH inhibited apoptotic cell death in the neurons, microglial cells, and endothelial cells (Additional file 1: Fig. S4). Consistently, taurine treatment significantly decreased the level of HOCl in the ischemic cortex but had no effect on the expression of MPO in the ischemic brains (Fig. 7A, B). Taurine treatment improved the neurological outcomes (Fig. 7C), reduced infarct volume (Fig. 7D, F), and attenuated the BBB permeability (Fig. 7E, G). The anti-apoptotic effects of taurine were further demonstrated, showing the downregulated Bax and the upregulated P62 and Bcl-2 in the ischemic brains (Fig. 6H, I). Furthermore, we investigated the expression of claudin-5, occludin and ZO-1, representative tight junctions for BBB permeability [43–45]. The taurine treatment rescued claudin-5, occludin and ZO-1 proteins in the ischemic brains (Fig. 7J, K). We also addressed the roles of HOCl in mediating endothelial monolayer disruption when bEND-3 cells were co-cultured with BV2 cells under OGD/R. The co-cultured system significantly increased HOCl production of bEND-3 cells (Additional file 1: Fig. S5A). FITC-dextran leakage test was used to detect the BBB permeability in the endothelial cells. Taurine treatment significantly alleviated the FITC-dextran leakage and reversed the endothelial monolayer’s barrier function (Additional file 1: Fig. S5B), inhibited the MMP-9 expression and claudin-5 degradation (Additional file 1: Fig. S5C–E). These results suggest that MPO-derived HOCl plays a crucial role in cerebral I/R injury.

### MPO/HOCl induces HMGB1 translocation and secretion in cerebral I/R injury

**Fig. 8:**
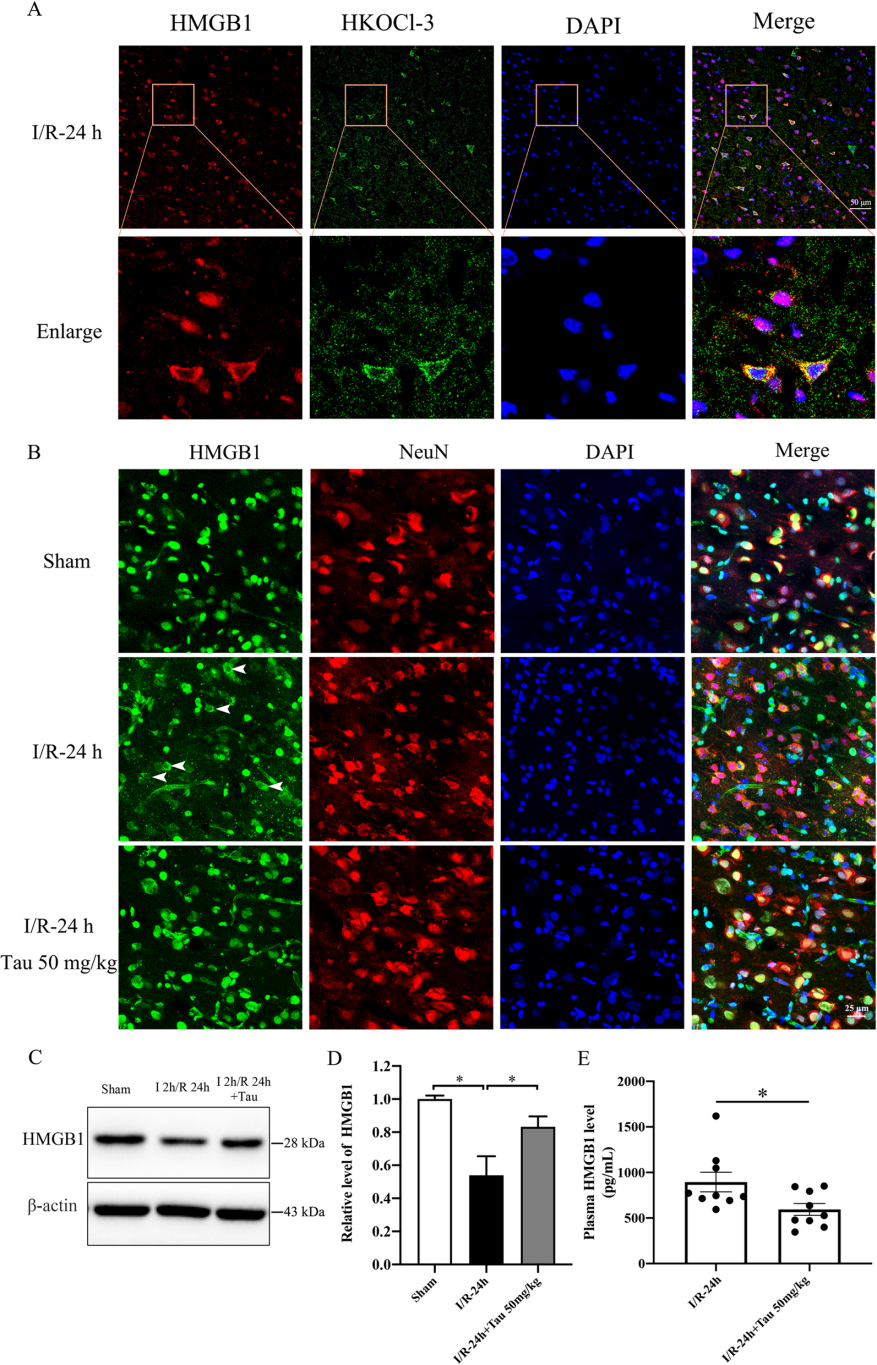
HOCl scavenger taurine inhibited HMGB1 secretion in rat ischemic brains during cerebral ischemia–reperfusion injury. I/R rats were subjected to 2 h of MCAO ischemia plus 24 h of reperfusion. Sham control rats were subjected to similar operations without MCAO. Taurine (Tau, 50 mg/kg) was intravenously administered at the onset of reperfusion. **A** Co-immunostaining detection of HOCl and HMGB1 location in I/R rat brains. **B** Immunofluorescent staining of HMGB1 in neurons of the ischemic cortical area. **C**, **D** Western blot and quantitative analysis for HMGB1 expression in I/R rat brains. **E** ELISA detection of HMGB1 secretion in rat plasma. *p < 0.05. All data are presented as mean ± SEM and each point denotes one animal. (Statistical methods: **D** one-way ANOVA followed by Tukey’s multiple comparisons test; **E** two-tailed *t*-test.)

We then investigated the effects of taurine, HOCl scavenger, on HMGB1 translocation and secretion in the rat ischemic brains in vivo. Immunofluorescent imaging revealed the cytoplasm colocalization of HMGB1 and HOCl (HKOCl-3 positive) in the ipsilateral side of the ischemic brains (Fig. 8A). Treatment of taurine significantly inhibited the translocation of HMGB1 to the cytoplasm of the neuronal cells in the ischemic brain tissues (Fig. 8B). Western blot analysis yielded consistent results with immunofluorescence. The level of HMGB1 was significantly decreased in the post-ischemic brains (Fig. 8C, D) but increased in the plasma (Fig. 8E), indicating the secretion of HMGB1. Compared with the I/R-24 h group, the taurine treatment group had significantly increased HMGB1 level in the ischemic brain but decreased HMGB1 level in the plasma (Fig. 8C–E). Taken together, those results directly demonstrate that MPO/HOCl could mediate the translocation and secretion of HMGB1 and aggravate brain damage and neurological deficits in cerebral I/R injury.

### Plasma HOCl level is correlated with infarct sizes and neurological deficits in acute ischemic stroke patients

**Fig. 9:**
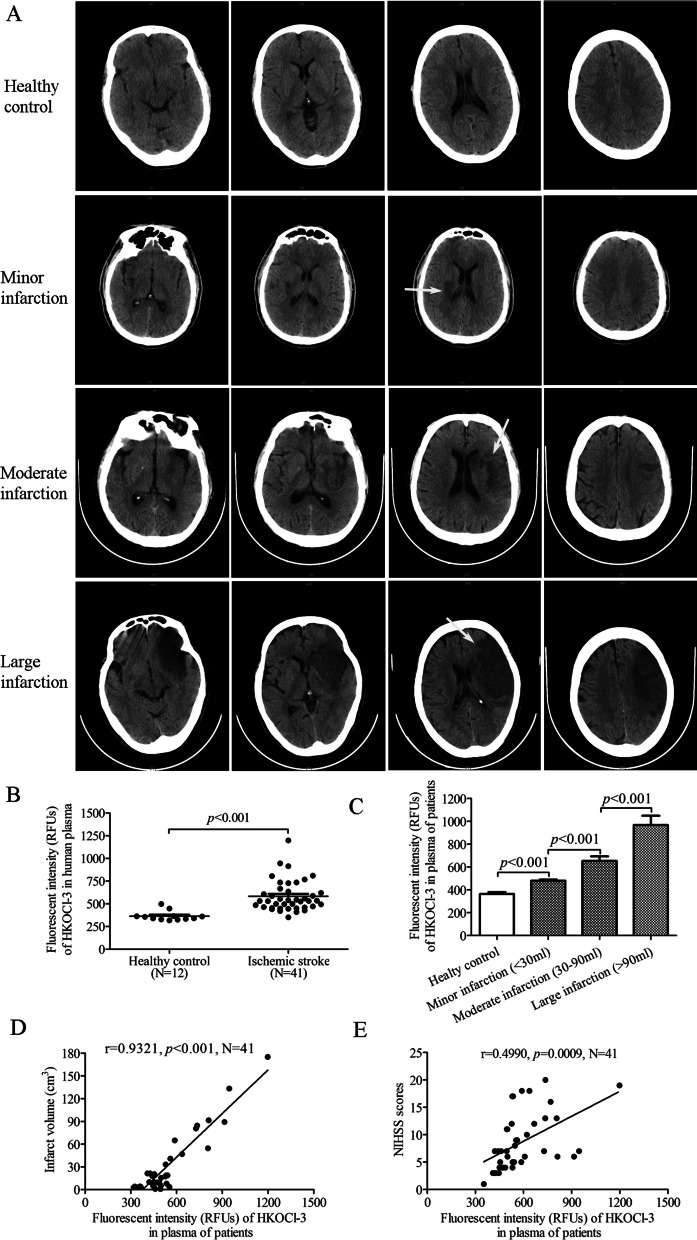
Plasma HOCl level was correlated with the severity of infarct volume and neurological deficit scores in ischemic stroke patients. **A** Representative CT scanning images showing the infarct area of the ischemic stroke patients (white arrow). **B** Using HKOCl-3 to detect plasma HOCl levels in ischemic stroke patients and healthy controls. **C** Plasma HOCl levels in different groups correspond to minor, moderate and large infarction sizes of ischemic stroke patients and healthy volunteers. **D** Pearson correlation coefficients of plasma HOCl level and infarct volume in acute ischemic stroke patients. **E** Pearson correlation coefficients of plasma HOCl level and NIHSS in acute ischemic stroke patients. All data are presented as mean ± SEM. (Statistical methods: **A** two-tailed *t*-test; **C** one-way ANOVA followed by Bonferroni multiple comparisons test; **D**, **E** Pearson correlation coefficient.)

We finally investigated the correlation of plasma HOCl level with infarct volume and neurological deficit scores in ischemic stroke patients. The infarct volume in the middle cerebral artery (MCA) territory of the ischemic stroke patient was measured by CT scanning (Fig. 9A). There was no significant difference in the baseline characteristics between the control and patient groups (Table 1). Blood samples were collected immediately after admission and plasma HOCl level was detected using HKOCl-3. Acute ischemic stroke patients had a significantly higher HOCl level in the plasma than the healthy control group (*p* < 0.001, Fig. 9B). The larger the infarct volume was, the higher the HOCl level detected (Fig. 9C). The increased HOCl concentration was positively correlated with the infarct volume (*r* = 0.9321, *p* < 0.001, Fig. 9D) and NIHSS (National Institutes of Health Stroke Scale) (*r* = 0.4990, *p* = 0.0009, Fig. 9E) in the ischemic stroke patients. These results suggest that the plasma HOCl level could be associated with the severity of brain damage in ischemic stroke patients.

**Table 1:** Summary of clinical data from controls and ischemic stroke patients

Clinical materials	Controls	Patients	*P* value
Gender (male/female)	7/5	16/13	0.853
Age (years)	58.00 ± 4.75	57.93 ± 2.86	0.989
Systolic pressure (mmHg)	140.75 ± 13.84	155.64 ± 7.14	0.301
Body temperature (℃)	36.56 ± 0.13	36.44 ± 0.10	0.448
Blood leukocyte (× 10^9^/L)	7.95 ± 0.68	7.15 ± 0.53	0.363
Blood neutrophils (× 10^9^/L)	5.05 ± 0.82	4.72 ± 0.38	0.682
Blood glucose (mmol/L)	5.74 ± 0.76	5.89 ± 1.78	0.856
Cholesterol (mmol/L)	5.31 ± 0.33	4.52 ± 0.30	0.096
LDL-C (mmol/L)	3.45 ± 0.22	3.19 ± 0.26	0.487
Hs-CRP (mg/L)	2.39 ± 1.33	2.69 ± 1.04	0.861

Data were presented as mean ± SEM

*LDL-C* low density lipoprotein cholesterol

*Hs-CRP* high sensitive C-reactive protein

## Discussion

The present study has following major discoveries: (1) PC12 cells had significantly increased levels of MPO/HOCl and aggravated apoptotic cell death when co-cultured with BV2 microglial cells after OGD/R exposure; (2) OGD/R exposure induced the release of MPO-containing exosomes from BV2 microglial cells that increased HOCl production and aggravated cell death in the transwell co-cultured PC12 cells; (3) HOCl production promoted disulfide HMGB1 formation and induced HMGB1 translocation and secretion; (4) the levels of MPO/HOCl were increased in neurons, endothelial cells and microglia of the ischemic brains during cerebral I/R injury; (5) MPO inhibitor 4-ABAH and HOCl scavenger taurine attenuated neurological deficits, infarct volume and the BBB disruption in cerebral I/R injury; (6) taurine inhibited HMGB1 translocation and secretion in the post-ischemic brains; (7) the induction of MPO/HOCl production increased infarct volume and the BBB disruption, and aggravates neurological deficits in cerebral I/R injury. (8) Plasma HOCl level was correlated with infarct sizes and neurological deficits in acute ischemic stroke patients. Based on our findings, we tentatively conclude that the MPO-containing exosomes released from activated microglia could increase HOCl production in adjacent cells, which may, in turn, mediate the translocation and secretion of disulfide HMGB1 in neurons, aggravating apoptotic cell death, the BBB disruption, and neurological deficits in cerebral I/R injury.

Neurons crosstalk with their surrounding cells continuously to maintain the development and function of the brain [46]. With the disturbance of the cerebral microenvironment, microglia could rapidly release soluble factors to bind with neuronal receptors, affecting neuronal cell functions. Microglia also release extracellular vesicles containing proteins, enzymes and mRNA to communicate with neurons [47]. Microglia release and secrete exosomes that are up-taken by target cells [5, 39, 48, 49]. The exosomes enter neighboring neurons to affect their functions in the ischemic brain [7]. Neutrophil-derived microparticles containing MPO could be transferred into vascular endothelial cells [50]. In the present study, we used transwell co-cultured PC12 cells and BV2 microglial cells for proof-of-the-concept that microglia could release MPO to affect neurological functions under OGD/R conditions. After exposed to OGD/R, the monocultured PC12 cells revealed unchanged MPO expression but had slightly increased HOCl level. The results indicate that the PC12 cells could produce a small amount of HOCl although the expression of MPO was undetectable. The inconsistency of MPO expression and HOCl production might be attributed to the different sensitivity of western blot and fluorescent imaging detection, respectively. Importantly, the levels of both MPO and HOCl were significantly increased in the PC12 cells when transwell co-cultured with BV2 cells after OGD/R challenge (Fig. 1A–D). However, when co-cultured with MPO-knockdown BV2 cells, the levels of MPO and HOCl in the PC12 cells were notably attenuated (Fig. 2E–G). These findings highlight the crucial role of BV2-derived MPO in promoting the production of MPO/HOCl within the PC12 cells in the transwell co-culture system. In addition, the OGD/R-treated BV2 cells released MPO-containing exosomes into the supernatant and increased HOCl production in the co-cultured PC12 cells. Furthermore, the incubation of the exosomes from the OGD/R-stimulated BV2 cells significantly increased HOCl production and apoptotic cell death in the PC12 cells. Treatments of MPO inhibitor 4-ABAH or HOCl scavenger taurine abolished the changes in the experiments. Those results suggest that microglial MPO-containing exosomes play crucial roles in mediating HOCl production and cell death in adjacent neuronal cells under OGD/R conditions. Interestingly, at the timepoint of 4 h OGD plus 6 h reoxygenation, although the BV2 cells had significantly upregulated MPO expression, the cell viability was unchanged when cultured with the PC12 cells, indicating co-cultured PC12 cells would not affect the survival of microglial cells under OGD/R conditions (Additional file 1: Fig. S6). Notably, in the in vivo ischemic brain sessions, both microglia and neurons revealed significantly increased MPO and HOCl levels after exposure to 2 h of MCAO plus 4 h of reperfusion. With the complex interactions of the microenvironment and circulating factors, due to technical burdens, we failed to obtain microglial MPO-containing exosomes from in vivo brain samples to support the results obtained from in vitro study. Since MPO/HOCl is often induced in stressed neurons either observed in this research (Fig. 1C, D, OGD slightly increased HOCl production in PC12 cells) or reported in other studies [51, 52]. In addition, during I/R injury, uptake of macrophage-derived exosomes triggers neuronal inflammatory cascade, resulting in exacerbated ROS accumulation in neurons [53]. Therefore, we have not exclusively ignored the possibility that microglia-derived exosomes may also trigger the expression of MPO/HOCl from PC12 cells themselves. Nevertheless, by integrating the in vitro and in vivo results, we logically interpret that the increased MPO/HOCl in the neurons could be at least in part attributed to the release of the MPO-containing exosomes from the microglia in post-ischemic brains. Subsequently, the induction of MPO-HOCl from the exosomes of microglia/macrophage (the Iba-1 positive cells) might contribute to brain damage and neurological deficits in ischemic stroke. For data interpretation, we should remark that the circulating peripheral inflammatory cells, such as infiltrated peripheral neutrophils and monocytes, could also aggravate brain damage in ischemic stroke [9, 54]. The infiltration of neutrophils was found in early phage ischemic brains to aggravate brain damages, while, the induction of monocytes was mainly presented in the ischemic brains at least 24 h later after ischemia onset and peaked at three days [55]. In addition, MPO could be also transported into sub-endothelium through endocytosis [56]. The roles of endocytosis in mediating MPO/HOCl production in the neurons remain to be further investigated. Thus, the MPO-derived HOCl production in the ischemic brains could be involved in multiple inflammatory cells, which remain to be further studied.

The translocation and release of HMGB1 from damaged neurons play crucial roles in triggering brain inflammatory response [57]. Oxidative stress promotes the translocation and release of HMGB1 [41, 42]. Our recent study indicates that peroxynitrite mediates HMGB1 secretion and induces the BBB disruption and hemorrhagic transformation in cerebral I/R injury [28]. HMGB1 can be either actively secreted by activated immune cells in oxidized form or passively released from necrotic cell death in reduced form. The passive release could be due to the disruption of the plasma membrane during cell death, while active secretion might be related to post-translational modifications of HMGB1 [58]. Whether HOCl mediates HGMB1 secretion remains unknown. In our results, the presence of HOCl aggravated the OGD/R-induced cell death and promoted the passive HMGB1 release in the PC12 cells. Meanwhile, HOCl treatment also induced the active secretion of HMGB1 from living PC12 cells although the integrity of plasma membranes was maintained under normoxic culture conditions. In the active secretion, HOCl increased the secretion of HMGB1 in the cytoplasm and supernatant. Immunofluorescence also showed that the level of HMGB1 was decreased in the nuclear but increased in the cytoplasm of the PC12 cells. Such changes are reversed by taurine treatment. Thus, we conclude that HOCl could promote both active secretion and passive release of HMGB1, subsequently aggravating cerebral I/R injury. In addition to MPO, we found that the microglia also released HMGB1 when co-cultured with PC12 cells under OGD/R conditions (Additional file 1: Fig. S7). As recent study revealed that inhibition of microglial HMGB1 release prevented neuroinflammation and protected hippocampal neuronal from type 2 diabetes mellitus [59], suggesting that microglia-derived HMGB1 functions as a pivotal mediator in modulating neurotoxic and pro-inflammatory activities. Nevertheless, this aspect requires more investigation.

HMGB1 is a redox-sensitive protein containing three conserved cysteine residues: Cys23, Cys45 and Cys106. The functions of HMGB1 depend on the redox state of its cysteines. Partially oxidized HMGB1 with a disulfide bond between Cys23 and Cys45, Cys106 in the thiol state to form disulfide HMGB1. The disulfide HMGB1 is essential for the active secretion and cytoplasmic translocation of HMGB1 [33] and activates immune cells to produce cytokines/chemokines [60]. HOCl has high reaction rates with cysteine thiols to oxidizing cysteines rapidly. The reaction of cysteine thiols with HOCl produces intermediate sulfinyl chloride (RS-Cl) to form the disulfides [61]. HMGB1 could affect redox state in ischemic brain injury [57]. Herein, we demonstrated that HOCl induced the formation of disulfide HMGB1 and promoted its active secretion. Although mass spectrometry is commonly used to detect the redox isoforms of HMGB1, western blot analysis provides convenient, cheap, and appropriate method to detect the disulfide HMGB1 [62–64]. Thus, we used western blot to investigate the HOCl-induced HMGB1 oxidation and secretion in vitro and in vivo*.* Upon HOCl stimulation, HMGB1 was oxidized to disulfide form, then translocated and secreted from the PC12 cells in vitro*.* The disulfide HMGB1 was significantly increased in the cytoplasm and the supernatant of the PC12 cells. HOCl was co-located with translocated HMGB1, suggesting the close correlation between HOCl production and HMGB1 translocation. Intramolecular disulfide bonds can make polypeptide chains more compact, thereby increasing the electrophoretic mobility under non-reducing conditions. Fully reduced HMGB1 with all-thiol cysteine could migrate to form a single band at the 28 kDa molecular weight. In contrast, disulfide HMGB1 would relatively be running fast and migrate to form a band at 26 kDa. Thus, in the western blot studies, there are two bands for HMGB1 and the lower one is disulfide HMGB1. Taken together, these results indicate that HOCl could promote HMGB1 cytoplasmic accumulation and active secretion by forming its disulfide bond form.

Furthermore, by using our newly developed high sensitive HKOCl-3 probe [38], we directly detected the production of HOCl in cerebral ischemia-reperfused rat models and acute ischemic stroke patients. Both MPO and HOCl were significantly increased in the microglia, neurons, and endothelial cells in ischemic rat brain tissues after exposed to 2 h of MCAO plus 4 h of reperfusion in vivo. HOCl was mainly produced and increased at the reperfusion stage after MCAO ischemia. Importantly, plasma HOCl level was positively correlated with brain damage and neurological deficits in both cerebral I/R injury rat model and acute ischemic stroke patients. Treatments of 4-ABAH or taurine attenuated apoptotic cell death and infarct volume, protected the BBB integrity and improved neurological functions in cerebral I/R injury. Notably, taurine can react with HOCl to produce taurine chloramine (TauCl) in activated neutrophils. In turn, TauCl inhibits nitric oxide and inflammatory mediators in macrophages [65]. As taurine has multiple bioactivities [66], we should be cautious in data interpretation. Thus, we use 4-ABAH as an MPO inhibitor in our study, which provides support evidence from different angles. With the series of experiments, we conclude that HOCl is a crucial player in BBB disruption and brain damage during cerebral I/R injury. The findings about the effect of HOCl on BBB disruption in cerebral I/R injury is consistent with previous study in which HOCl production contributes to LPS-mediated BBB breakdown in vivo and in vitro [54]. The production of HOCl from chlorinated inflammatory mediators induce potent lipotoxic responses in brain endothelial cells and impact inflammation-induced BBB dysfunction [67, 68]. Therefore, MPO/HOCl could be critical therapeutic targets for ischemic stroke.

**Fig. 10:**
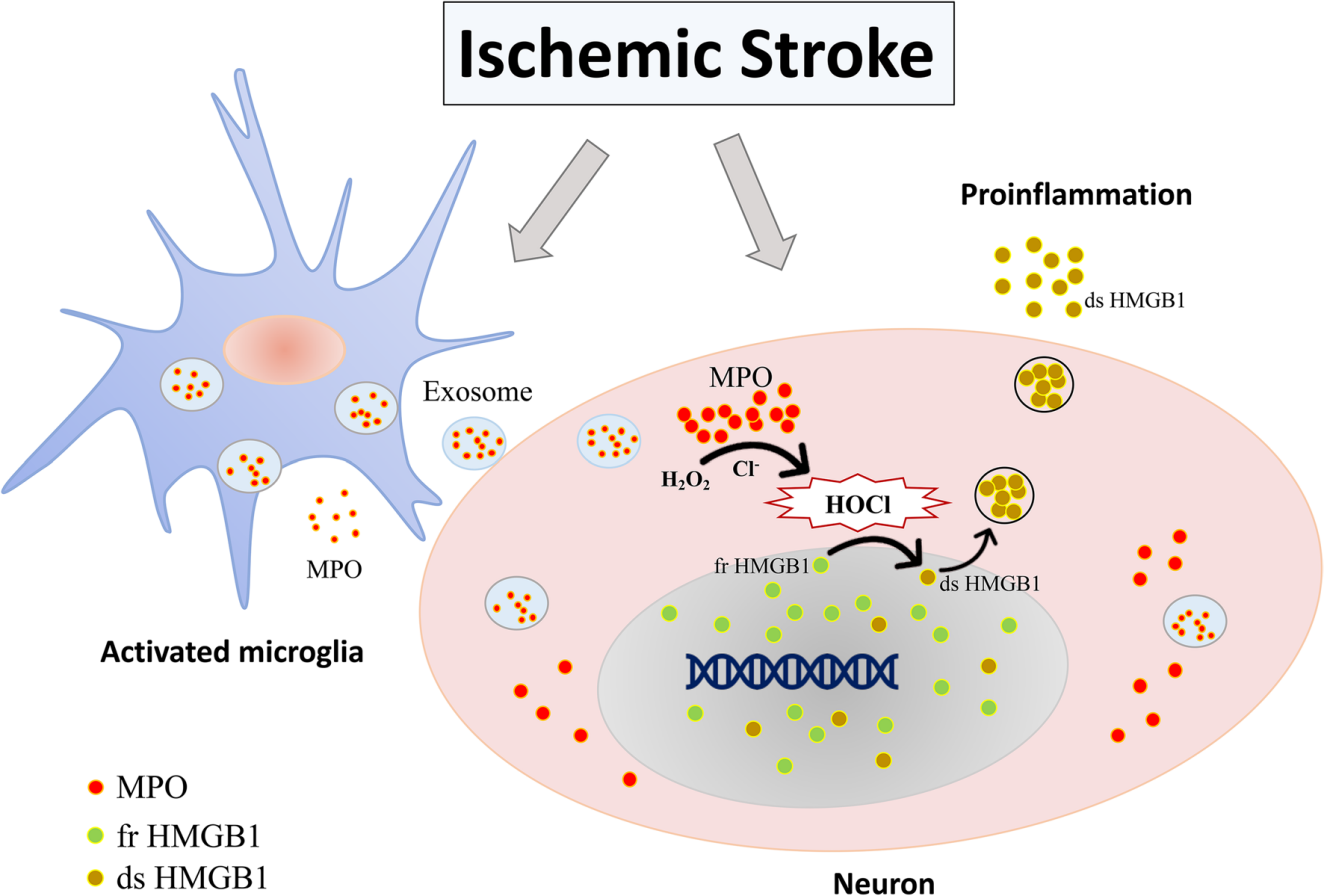
Schematic diagram showing that microglia-derived myeloperoxidase (MPO) can be transferred into neuron through exosomes, increasing the MPO and HOCl production in neuron. HOCl facilitates active cytoplasmic translocation and secretion of HMGB1 in the disulfide form, promoting inflammation and aggravating brain damage

In conclusion, the MPO-containing exosomes released from microglia could induce HOCl production and aggravate adjacent neuronal cell death in cerebral I/R injury. Furthermore, plasma HOCl level could be a novel biomarker for indexing brain damage in ischemic stroke patients (Fig. 10).

## Material and methods

### Correlation analysis of plasma HOCl level and infarct sizes and neurological deficits in acute ischemic stroke patients

We performed a clinical study at Sun Yat-sen Memorial Hospital, Sun Yat-sen University. The protocol was approved by the medical ethics committee of the university (No. SYSEC-KY-KS-059). All patients have been informed of the background and procedures of the study and signed the informed consent before enrollment. First-ever stroke patients aged 40–80 years old were included regardless of gender. The recruitments of ischemic stroke patients had the following inclusive criteria: (a) having a primary confirmed diagnosis of ischemic stroke caused by the MCAO after 6–72 h of symptoms onset (NIHSS > 0 and CT/MRI detected acute infarct area); (b) having competent to give written informed consent. The exclusion criteria for the patients include (a) with cancer; (b) with transient ischemic attack (TIA) or intracranial hemorrhage or subarachnoid hemorrhage (SAH) within 6 months; (c) with hepatic, renal, hematologic diseases; (d) with mental disorder, serious dementia; (e) with major surgery in the previous 6 months; (f) with pregnancy; (g) with epilepsy. Venous blood samples were obtained from the recruited patients immediately at admission and temporarily stored at 4 °C in EDTA-K2 coated tubes (aged between 40–80 years, *N* = 41). The NIHSS [69] was routinely used to evaluate the neurological deficits of each patient at admission by an investigator who was blinded to the experimental setting. The infarct volume was determined by the manual tracing technique on CT scanning image as previously described [70, 71]. The basic clinical data including body temperature, blood leukocyte, highly sensitive C-reactive protein (Hs-CRP) were also collected to exclude patients with symptoms of peripheral infection that might affect plasma HOCl level. Healthy individuals aged between 40–80 years without any parenchymal organ ischemia or inflammatory disease in the past three months were recruited as the control group (matched with similar ages and sex, *N* = 12).

All blood samples were assayed with HKOCl-3 probe immediately upon collection (< 60 min) to minimize the degradation of HOCl ex vivo. Briefly, blood samples were centrifuged at 1800*g* for 10 min at 4 °C, then the extracted plasma was co-incubated with the HKOCl-3 probe (2 μM) at 37 °C in darkness for 30 min and then examined using a multimode reader (SpectraMax M5, Molecular Devices). An equal volume of PBS was taken as a negative control. Each experiment was performed independently at least three times. All results were adjusted by subtracting the negative control to avoid background interference.

### Animals

Animals were obtained from the Laboratory Animal Unit, the Faculty of Medicine, the University of Hong Kong, which is an AAALAC International accredited service unit. Animal experiments were approved by the Committee on the Use of Live Animals in Teaching and Research, the University of Hong Kong (CULATR number: 5062-19). All animals were housed in 14-h day/light cycles with free feeding. All the studies followed ARRIVE guidelines and the Guide for the Care and Use of Laboratory Animals of the National Institutes of Health.

### Cerebral I/R injury and drug treatment

Male Sprague–Dawley (SD) rats were randomly assigned into groups: sham-operation (sham), cerebral I/R, I/R plus 4-ABAH (I/R + 4-ABAH), I/R plus taurine (I/R + taurine) and sham**-**operation plus 4-ABAH (4-ABAH). We adopted a transient MCAO model to mimic ischemic stroke as previously described with modification [72]. Briefly, male SD rats (250–300 g) were anesthetized with 10% chloral hydrate. An MCAO monofilament (Beijing Cinontech Co., Ltd; China; 2838-A4) was inserted into the internal carotid artery via the external carotid artery until mild resistance. After 2 h of ischemia, the monofilament was withdrawn to allow reperfusion. The success of the MCAO model was confirmed by 2,3,5-triphenyltetrazolium chloride (TTC) staining. Sham control rats were subjected to similar MCAO operation procedures but without occlusion. MPO-specific inhibitor 4-ABAH (20 mg/kg, Sigma, 5351-17-7) was intraperitoneally injected at the onset and 12 h of reperfusion, respectively (I/R + 4-ABAH group) [8]. Taurine (50 mg/kg, Aladdin, T103831, China) was intravenously administered at the onset of reperfusion (I/R + taurine). An equal volume of normal saline was used to replace 4-ABAH or taurine in the I/R group.

### Behavioral test

A modified neurological severity score (mNSS) was performed at 24 h of reperfusion to evaluate the neurological deficit of the rats as previously described [73]. The mNSS is a composite of motor, sensory, reflex, and balance tests, graded on a scale of 0 to 18 (normal score, 0; maximal deficit score, 18).

### Infarct volume

Infarct volume was measured at 24 h of reperfusion or at the same time points of sham operation. The rat brain was removed and sliced into 2.0-mm-thick slices by a brain sectioning matrix (Beijing Cinontech Co., Ltd; China; 300–600 g). Coronal brain slices were incubated with 2% TTC (Sigma, 298-96-4) at 37 °C for 20 min. The stained slices were photographed and the area of infarct in each slice was evaluated using the software Image-Pro Plus 6.0 (Media Cybernetics, USA). The percentage of infarct volume was measured according to a previously described method [74]: infarct volume (%) = [(volume of the contralateral hemisphere – the red volume of the intact ipsilateral hemisphere)/ 2 × contralateral hemisphere volume] × 100%.

### BBB integrity

The BBB leakage was assessed by Evans blue (EB) assay as previously reported [72]. The rats were intravenously administered with 2% EB solution (4 mg/kg, Biosharp) through the femoral vein at 24 h of reperfusion or sham operation. Two hours later, the rats were killed by cardiac perfusion with PBS. After rat brains were removed, the ischemic hemispheres were weighed and homogenized in 50% trichloroacetic acid. The samples were then incubated at 4 °C in darkness overnight and centrifuged at 12,000 *g* for 30 min. The supernatant was spectrophotometrically quantified at 620 nm wavelength (SpectraMax M5, Molecular Devices) to determine the amount of extravasated EB dye.

### Immunofluorescence

Brains were removed and fixed with 4% paraformaldehyde at 4 °C overnight. Frozen sectioning was performed after sucrose gradient dehydration followed by blocking with 10% goat serum containing 0.5% Triton X-100 at room temperature for 30 min. Brain sections were incubated with different primary antibodies at 4 °C overnight, including anti-MPO (Abcam, ab90812, 1: 1000), anti-NeuN (Abcam, ab177487, 1: 1000), anti-CD31 (Abcam, ab222783, 1: 1000), anti-Iba-1 (Abcam, ab153696, 1: 1000), anti-GFAP (Abcam, ab7260, 1: 1000) antibodies. The sections were washed with PBS 3 times and incubated with the mixture of Alexa Fluor 488 conjugated goat-anti-mouse IgG (Beyotime, A0428, 1: 500) or Alexa Fluor 555 conjugated donkey-anti-rabbit IgG (Beyotime, A0453, 1: 500) for 1 h in darkness at room temperature, followed by staining with DAPI for 5 min. After being washed with PBS 3 times, sections were mounted with fluorescent mounting medium (Dako, S3023), and examined by a confocal laser scanning microscope (LSM 780, Carl Zeiss).

### TUNEL assay

Brain-frozen sections were incubated with the primary antibodies at 4 °C overnight as described above, including anti-NeuN, anti-CD31 or anti-Iba-1. The sections were washed 3 times with PBS and then incubated with the TUNEL reaction mixture (Roche, 11684795910) and Alexa Fluor 555 conjugated donkey-anti-rabbit IgG for 1 h at room temperature, followed by staining with DAPI for 5 min. After washing with PBS, sections were mounted with fluorescent mounting medium (Dako, S3023), and then examined by using a confocal laser scanning microscope (LSM 780, Carl Zeiss). The number of apoptotic cell death was calculated by counting the TUNEL-positive cells per mm^2^ from all images taken in the same cortical regions with HKOCl-3 staining sessions. Six images per region and five sections per brain were taken according to a similar experimental method [75]. Cell counting was carried out by an investigator who was blinded to the experimental designs.

### HOCl detection

We determined the level of HOCl by using HKOCl-3, a novel fluorescent probe with high selectivity and sensitivity and a rapid turn-on response [38]. In cellular experiments, the cultured cells were co-incubated with HKOCl-3 (2 μM) in 12-well plates in dark for 30 min. After washing with PBS, the samples were mounted, and the fluorescent image was observed and taken under a fluorescent microscope (l × 71, OLYMPUS). In animal experiments, the blood samples were obtained from femoral veins before the animals were killed and the plasma HOCl level was detected by using similar methods as human blood samples. Then, brain tissues were collected for fresh frozen sections, and brain slices were incubated with HKOCl-3 (10 μM) at 4 °C in darkness for 30 min, followed by staining with DAPI for 5 min. After washing with PBS 3 times, the sections were mounted with fluorescent mounting medium (Dako, S3023) and examined by a fluorescent microscope (BX51, OLYMPUS). Cell counting was carried out by an investigator blinded to the experimental settings.

### Preparation and quantification of HOCl

HOCl was prepared by the dilution of the concentrated stock solution of sodium hypochlorite. We determined the concentration of HOCl by using the iodometric titration method.

### Cell culture

BV2 microglia, PC12 neuronal cells and brain microvascular endothelial bEND-3 cells were purchased from Shanghai Aolu Biotechnology Co., Ltd, China. BV2 cells were cultured in DMEM/ F12 medium (Gibco, 11330-032) supplemented with 10% fetal bovine serum (FBS, Gibco, 10099-141) and 1% penicillin/streptomycin (HyClone, SV30010). PC12 or bEND-3 cells were cultured in DMEM medium (HyClone, SH30243.01) supplemented with 10% FBS and 1% Penicillin/ Streptomycin. Before treatment with HOCl, the medium was removed and washed with Hanks’ buffered salt solution (HBSS) to prevent reactions of the medium with the HOCl. PC12 cells were then exposed to different concentrations of HOCl (0–100 μM) diluted in HBSS for 15 min.

### MPO-knockdown BV2 cell line construction

The shRNA against mouse MPO was purchased from the Guangzhou IGE biotechnology Co., Ltd (Guangzhou, China) to stably knock down MPO in BV2 cells. Briefly, the sequence of shRNA was annealed and ligated into a pLKO.1 lentiviral vector. A scrambled shRNA was used as a control. Lentiviral particles were prepared by co-transfecting HEK293T cells with a package plasmid containing the MDLg, MD2g and RSV genes and shRNA vectors using Lipofectamine 2000 reagent according to the manufacturer’s instructions. After 48 and 72 h transfection, the medium was collected, and the supernatant was used to infect the BV2 cells with polybrene. Two days after infection, the cells were selected with 2.5 μg/ml puromycin until no cells died, and knockdown efficiency of MPO in the cells was evaluated by western blot.

### Transwell co-cultured cell systems

For the co-culture experiments, we used a transwell culture system which mimicked the in vivo conditions as previously described [76]. Briefly, PC12 cells (2.5 × 10^5^ cells/well) were plated in 6-well dishes or inserts. The BV2 cells (1.5 × 10^5^ cells/well) were plated in the transwell inserts on top of the wells or in the wells. The co-cultured cell systems were incubated in DMEM/F12 medium supplemented with 10% FBS and 1% penicillin/streptomycin. For comparison, the same densities of PC12 cells were plated without transwell chambers.

### Oxygen–glucose deprivation/ reoxygenation (OGD/R) and drug treatment

Cells seeded in 6, 12 or 96-well plates or the co-cultured transwell systems were incubated with glucose-free DMEM medium (Gibco, 11966-025) in a hypoxic incubator (MIC-101, Billups-Rothenberg Inc.) containing the gas mixture of 95% N_2_ and 5% CO_2_ for 4 h at 37 °C, and then returned to normal culture conditions. 4-ABAH (50 μM), taurine (2 mM) or PBS was added into the medium at the onset of reoxygenation. For the normal control group, cells were incubated under NC without OGD/R exposure.

### Exosome isolation and labeling

After 4 h of OGD, the BV2 cells were incubated with refreshed DMEM plus 10% exosome-depleted FBS (Gibco, A2720803) and 1% penicillin/streptomycin for 24 h. We isolated exosomes from the cultured medium using ultracentrifugation according to previous studies [77]. Briefly, the medium was collected after the cells were exposed to 24 h of reoxygenation and centrifuged at different speeds. Firstly, the supernatant was collected and centrifuged at 300 *g* at 4 °C for 10 min to remove living cells. Then, the dead cells and cell debris were sequentially removed by centrifugation at 3000 *g* and 10,000 *g* for 30 min at 4 °C. Followed by filtering through a 0.22 μm membrane (Merck Millipore), the supernatant was transferred to a 70 ml ultracentrifuge tube (Beckman, 355622) and ultracentrifuged at 100,000 *g* (Beckman, Ti70) for 70 min at 4 °C. The bottom pellet was resuspended in PBS and collected by another ultracentrifugation at 100,000 for 70 min. Finally, the exosome pellet was dissolved in 100 μL PBS for further studies.

Isolated exosomes were labeled with CM-Dil, a fluorescent dye. Briefly, exosomes were incubated with 20 μM CM-Dil for 15 min with regular mixing. Excess dye from the labeled sample was removed by washing 3 times with PBS. After purifying and labeling, the Dil-labeled exosomes were co-cultured with PC12 cells for 4 h at 37 °C. Then PC12 cells were washed with PBS and fixed in 4%PFA. The uptake was observed by fluorescence microscopy.

### Western blot analysis

Samples from the cultured cells or fresh brain tissues were prepared for protein lysates using total protein lysis buffer (Beyotime, P0013) and analyzed by SDS-PAGE or non-reducing SDS-PAGE. The membrane was incubated with primary antibodies against HMGB1 (Abcam, ab79823, 1:1000, 25 kDa), MPO (Abcam, ab208670, 1: 1000, 59 kDa), CD63 (Abcam, ab216130, 1:1000, 26 kDa), MMP-2 (Abcam, ab92536, 1: 1000, 65 kDa), MMP-9 (Abcam, ab76003, 1: 1000, 92 kDa latent and 83 kDa active form), Claudin-5 (Abcam, ab15106, 1: 1000, 24 kDa) and GADPH (Cell Signaling Technology, #2118S, 1: 5000, 37 kDa) at 4 °C overnight, followed by incubation with anti-rabbit or anti-mouse IgG (MultiSciences (LiankeBio), GAR007, 1:5000) for 1 h at room temperature. The immune bands were visualized using the ECL kit (KeyGEN BioTECH, KGP1126) and photographed with ChemiDoc XRS + (Bio-Rad, Hercules, CA, USA). Each experiment was performed independently 5 times.

### MTT assay

We used a 3-(4,5)-dimethylthiahiazo (-z-y1)-3,5-diphenytetrazoliumromide (MTT) kit (Sigma, M5655) to detect cell viability. Briefly, cells were plated in 96-well dishes and incubated with MTT solution (final concentration: 0.5 mg/ ml) at 37 °C in darkness for 4 h followed by dimethyl sulfoxide (DMSO, 150 μl/ well). Dishes were gently stirred in a gyratory shaker for 5 min. The O.D. value was examined by a multimode reader (SpectraMax M5, Molecular Devices) at a wavelength of 490 nm. Each experiment was performed independently 5 times.

### Flow cytometry

We used the Annexin V-FITC/PI apoptosis detection kit (KeyGEN BioTECH, KGA107) to examine apoptotic cell death. Briefly, isolated single-cell suspensions were surface-stained with Annexin-V and PI at room temperature in darkness for 15 min. The cell populations were determined by flow cytometry (LSR II, BD). Each experiment was performed independently 5 times.

### Nuclear/cytosolic fractionation

Cytoplasmic localization of HMGB1 was detected by density fractionation of cytoplasmic homogenates. Resting or HOCl-stimulated PC12 cells in 6-well plates were harvested. Nuclear and cytosolic extractions were collected by using Nuclear and Cytoplasmic Protein Extraction kit (Thermo Scientific, 78835) according to the manufacturer’s protocols.

### Statistical analysis

Image Pro Plus 6.0 (Media Cybernetics, lnc., USA) software was used to analyze the optical density of Western blot’s results and calculate the number of fluorescence-positive cells. Statistical analysis was performed with SPSS 19.0 (SPSS Inc., USA) software. Data were presented as means ± SEM. Pearson correlation coefficients were used to analyze the correlation of the plasma HOCl level with NIHSS as well as infarct volume in the patients. Independent *t*-test or Pearson Chi-square test was used to compare the difference between the two groups. Kruskal–Wallis test was used for multiple comparisons among the data from HKOl-3 positive cells counting without the homogeneity of variance. Two-way ANOVA was used to compare the results among multiple groups from flow cytometry assay followed by Bonferroni's post hoc test. One-way ANOVA was used to compare the results among multiple groups from other assays followed by Bonferroni's post hoc test. *P* < 0.05 was considered statistically significant. Statistical tests were two-tailed.

### Supplementary Information

**Additional file 1: Figure S1.** The increased MPO expression in transwell-PC12 cells started at 6 h of reoxygenation and continued to 12 h and 24 h of reoxygenation. Western blot analysis for MPO expression in PC12 cells under monoculture or co-culture with BV2 cells conditions after 4 h of OGD and 6 h, 12 h and 24 h of reoxygenation. Transwell-PC12 cells: PC12 and BV2 co-cultured cells were exposed to 4h OGD with 6h, 12h or 24h of reoxygenation. **Figure S2.** Immunofluorescent imaging of HMGB1 location in HOCl-treated primary neuron cells. The primary cells were subjected to HOCl treatment (50 μM, dissolved in HBSS) for 15 min and re-incubated in normal cultured medium for 2 h or 24 h. HGMB1, green fluorescence; DAPI, blue fluorescence; Nestin, red fluorescence. HOCl promoted the nuclear-to-cytoplasmic translocation of HMGB1 in primary cultured neurons. **Figure S3.** Detections of MPO and HOCl in astrocyte after I/R injury. (A) The fluorescent co-staining of MPO and GFAP in the ischemic hemisphere of MCAO rats at24 h after reperfusion, bar = 50 μm. (B) The purity of primary astrocytes shown by fluorescent staining of GFAP, bar = 200 μm. (C) Detection of HOCl in primary astrocytes after OGD/R with HKOCl-3 probe, bar = 200 μm. **Figure S4.** Effects of 4-ABAH on apoptotic cell death in the microglia, neurons and brain endothelial cells after I/R injury. (A) Co-location of TUNEL staining and Iba-1 in microglia of the ischemic cortical area; Bar=100 μm. (B) Co-immunostaining detection of TUNEL and NeuN for identifying neuronal apoptotic cell death in the ischemic cortical area; Bar=100 μm. (C) Co-immunostaining detection of TUNEL and CD31 for identifying apoptotic cell death in microvascular endothelial cells of the ischemic cortical area; Bar=100 μm; **p< 0.01. All data are presented as Mean ± SEM. (Statistic methods: two-tailed *t*-test). **Figure S5.** Effect of HOCl on endothelial permeability and expression of MMP-9 and claudin-5 in bEND-3 cells when co-cultured with BV2 under 4 h OGD plus 24 h reoxygenation condition in vitro. In the taurine treatment group, the normal medium with taurine (2 mM) was added at the onset of reoxygenation. (A) HKOCl-3 staining HOCl production from PC12 cells in single cultured plates or co-cultured transwells with BV2 cells under NC or OGD/R condition, bar = 200 μm. (B) In the upper chamber, bEND3 cells were grown on the insert with assay medium containing FITC-dextran. In the lower chamber, BV2 cells were cultured at the plate and endothelial permeability was assessed by measuring the clearance of FITC-dextran from the upper chamber to the lower chamber. (C) In the upper chamber, BV2 cells were cultured in the insert; In the lower chamber, bEND3 cells were cultured at the plate. Western blot analysis detected the expression of MMP-9 and claudin-5 in bEND-3 cells co-cultured with BV2 cells in a transwell co-culture system. (D, E) Quantitative analysis of MMP-9 and claudin-5 expression; N=3. Transwell-OGD/R: BV2 and PC12 co-cultured cells were exposed to 4 h OGD with 24 h of reoxygenation. *p< 0.05, **p< 0.01. All data are presented as Mean ± SEM. (Statistic methods: one-way ANOVA followed by Tukey’s multiple comparisons test). **Figure S6.** MTT assay for cell viability in BV2 cells co-cultured with PC12 cells under NC or OGD/R conditions after 4 h of OGD and 0 h or 6 h of reoxygenation. Transwell-NC: co-culture BV2 and PC12 cells under normal culture condition; Transwell-0h, 6h: BV2 and PC12 co-culture cells were exposed to 4 h OGD with 0 h or 6 h of reoxygenation. ****p< 0.0001. All data are presented as Mean ± SEM. (Statistic methods: one-way ANOVA followed by Tukey’s multiple comparisons test). **Figure S7.** Western blot analysis for HMGB1 expression in BV2 cells under monoculture or co-culture with PC12 cells conditions after 4 h of OGD and 24 h of reoxygenation. Transwell-OGD/R: BV2 and PC12 co-cultured cells were exposed to 4h OGD with 24h of reoxygenation. **p< 0.01. All data are presented as Mean ± SEM. (Statistic methods: one-way ANOVA followed by Tukey’s multiple comparisons test).

## Data Availability

The datasets used and/or analyzed during the current study are available from the corresponding author upon reasonable request.

## Abbreviations

BBB: Blood–brain barrier
CNS: Central nerve system
DAMPs: Damage-associated molecular patterns
EB: Evans blue
EVs: Extracellular vesicles
HBSS: Hanks’ buffered salt solution
HMGB1: High-mobility group box 1
HOCl: Hypochlorous acid
Hs-CRP: Highly sensitive C-reactive protein
HUVECs: Human umbilical vein endothelial cells
I/R: Ischemia/reperfusion
MCAO: Middle cerebral artery occlusion
mNSS: Modified neurological severity score
MPO: Myeloperoxidase
NC: Normoxic conditions
NVUs: Neurovascular units
OGD/R: Oxygen–glucose deprivation and reoxygenation
ROS: Reactive oxygen species
SAH: Subarachnoid hemorrhage
TauCl: Taurine chloramine
TBI: Traumatic brain injury
TEM: Transmission electron microscope
TIA: Transient ischemic attack
TTC: 2,3,5-Triphenyltetrazolium chloride
