# Supplementary material for: Hypochlorous acid derived from microglial myeloperoxidase could mediate high-mobility group box 1 release from neurons to amplify brain damage in cerebral ischemia–reperfusion injury
Source: J Neuroinflammation. 2024 Mar 21;21:70. doi: 10.1186/s12974-023-02991-8 (PMC10958922; doi:10.1186/s12974-023-02991-8)
Supplement: Supplementary file 1 — Additional file 1: Figure S1. The increased MPO expression in transwell-PC12 cells started at 6 h of reoxygenation and continued to 12 h and 24 h of reoxygenation. Western blot analysis for MPO expression in PC12 cells under monoculture or co-culture with BV2 cells conditions after 4 h of OGD and 6 h, 12 h and 24 h of reoxygenation. Transwell-PC12 cells: PC12 and BV2 co-cultured cells were exposed to 4h OGD with 6h, 12h or 24h of reoxygenation. Figure S2. Immunofluorescent imaging of HMGB1 location in HOCl-treated primary neuron cells. The primary cells were subjected to HOCl treatment (50 μM, dissolved in HBSS) for 15 min and re-incubated in normal cultured medium for 2 h or 24 h. HGMB1, green fluorescence; DAPI, blue fluorescence; Nestin, red fluorescence. HOCl promoted the nuclear-to-cytoplasmic translocation of HMGB1 in primary cultured neurons. Figure S3. Detections of MPO and HOCl in astrocyte after I/R injury. (A) The fluorescent co-staining of MPO and GFAP in the ischemic hemisphere of MCAO rats at24 h after reperfusion, bar = 50 μm. (B) The purity of primary astrocytes shown by fluorescent staining of GFAP, bar = 200 μm. (C) Detection of HOCl in primary astrocytes after OGD/R with HKOCl-3 probe, bar = 200 μm. Figure S4. Effects of 4-ABAH on apoptotic cell death in the microglia, neurons and brain endothelial cells after I/R injury. (A) Co-location of TUNEL staining and Iba-1 in microglia of the ischemic cortical area; Bar=100 μm. (B) Co-immunostaining detection of TUNEL and NeuN for identifying neuronal apoptotic cell death in the ischemic cortical area; Bar=100 μm. (C) Co-immunostaining detection of TUNEL and CD31 for identifying apoptotic cell death in microvascular endothelial cells of the ischemic cortical area; Bar=100 μm; **p< 0.01. All data are presented as Mean ± SEM. (Statistic methods: two-tailed t-test). Figure S5. Effect of HOCl on endothelial permeability and expression of MMP-9 and claudin-5 in bEND-3 cells when co-cultured with BV2 under 4 h [file 12974_2023_2991_MOESM1_ESM.pdf]

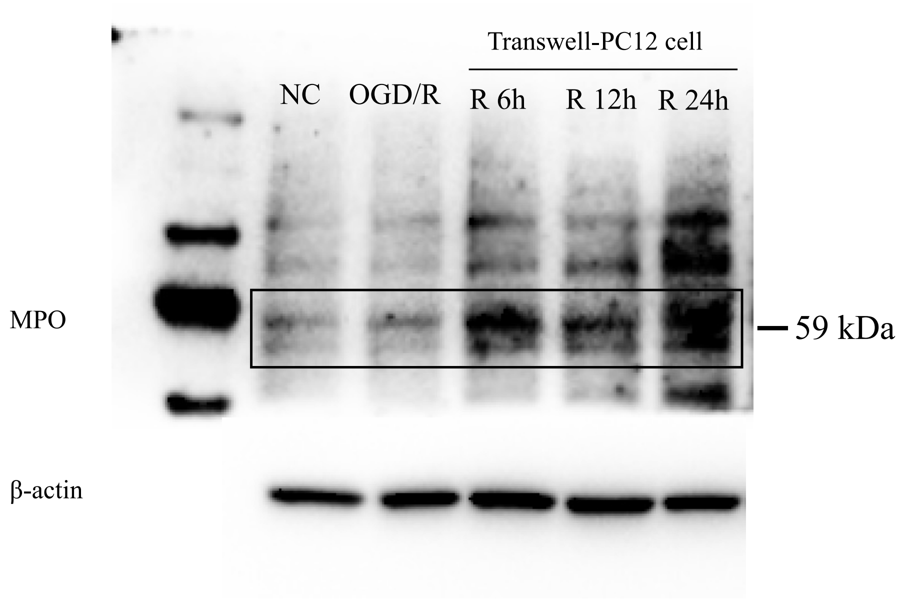

Figure S1: The increased MPO expression in transwell-PC12 cells started at 6 h of reoxygenation and continued to 12 h and 24 h of reoxygenation. Western blot analysis for MPO expression in PC12 cells under monoculture or co-culture with BV2 cells conditions after 4 h of OGD and 6 h, 12 h and 24 h of reoxygenation. Transwell-PC12 cells: PC12 and BV2 co-cultured cells were exposed to 4h OGD with 6h, 12h or 24h of reoxygenation.

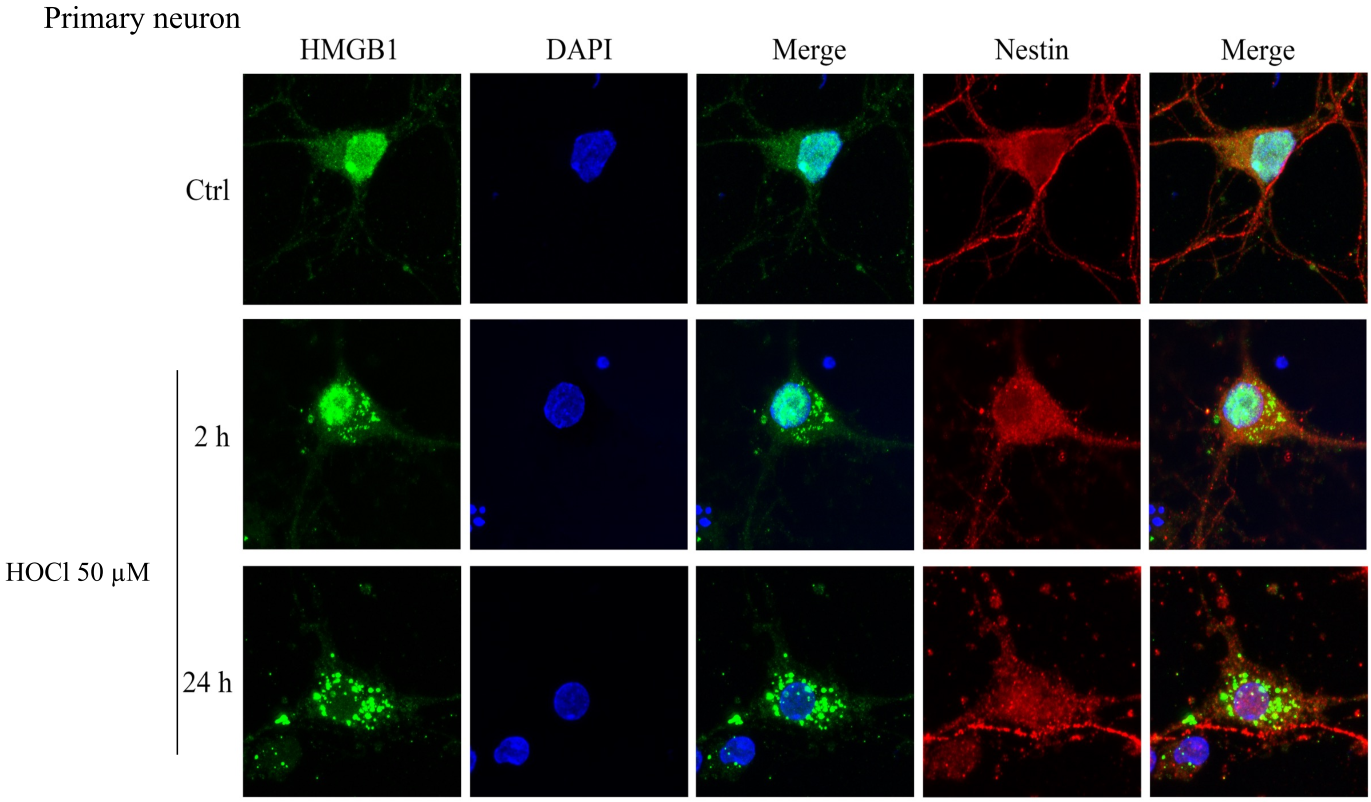

Figure S2: Immunofluorescent imaging of HMGB1 location in HOCl-treated primary neuron cells. The primary cells were subjected to HOCl treatment (50 μM, dissolved in HBSS) for 15 min and re-incubated in normal cultured medium for 2 h or 24 h. HGMB1, green fluorescence; DAPI, blue fluorescence; Nestin, red fluorescence. HOCl promoted the nuclear-to-cytoplasmic translocation of HMGB1 in primary cultured neurons.

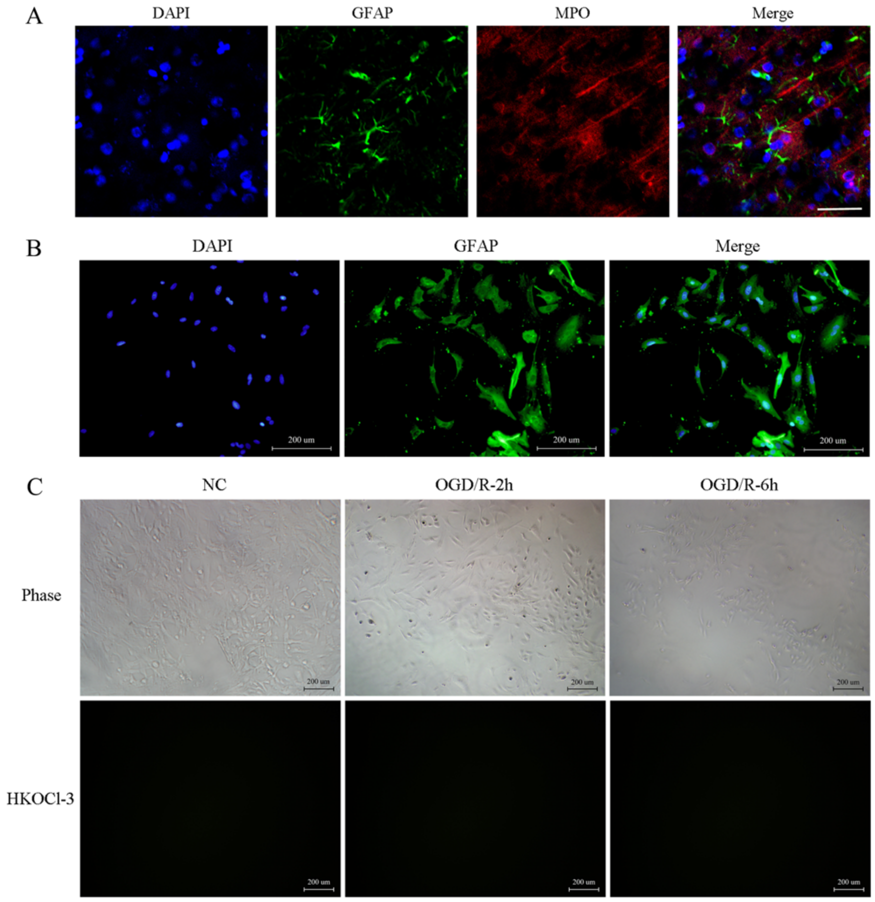

Figure S3: Detections of MPO and HOCl in astrocyte after I/R injury. (A) The fluorescent co-staining of MPO and GFAP in the ischemic hemisphere of MCAO rats at24 h after reperfusion, bar = 50 μm. (B) The purity of primary astrocytes shown by fluorescent staining of GFAP, bar = 200 μm. (C) Detection of HOCl in primary astrocytes after OGD/R with HKOCl-3 probe, bar = 200 μm.

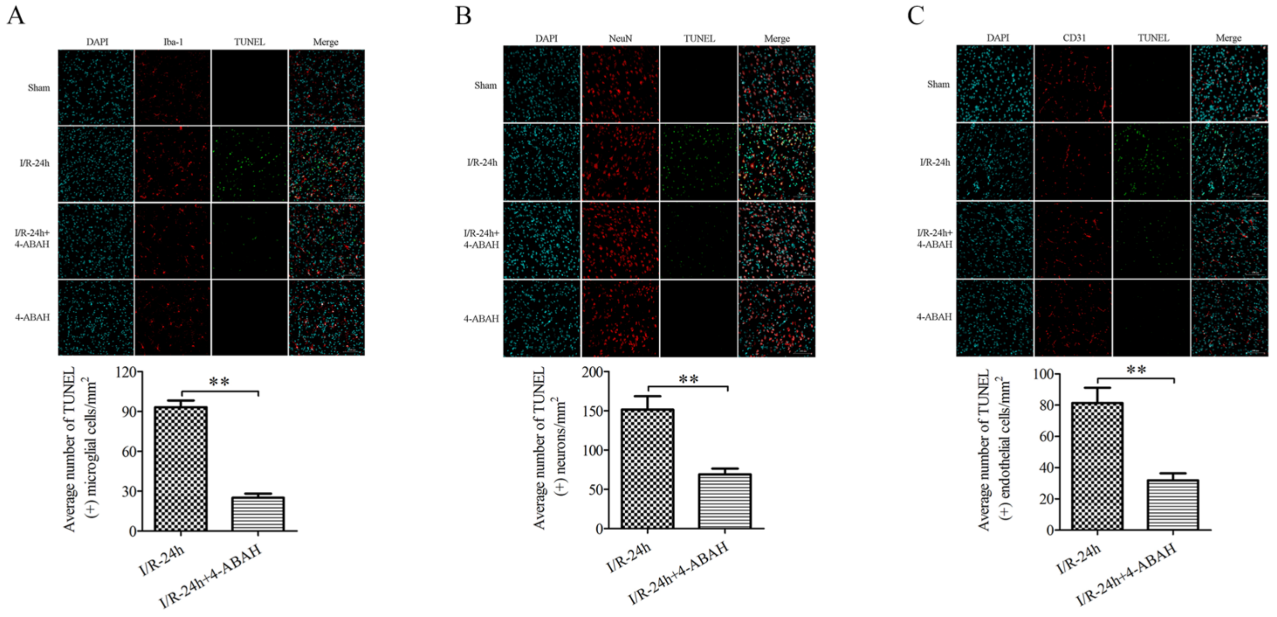

Figure S4: Effects of 4-ABAH on apoptotic cell death in the microglia, neurons and brain endothelial cells after I/R injury. (A) Co-location of TUNEL staining and Iba-1 in microglia of the ischemic cortical area; Bar=100 µm. (B) Co-immunostaining detection of TUNEL and NeuN for identifying neuronal apoptotic cell death in the ischemic cortical area; Bar=100 µm. (C) Co-immunostaining detection of TUNEL and CD31 for identifying apoptotic cell death in microvascular endothelial cells of the ischemic cortical area; Bar=100 µm; $^{**}p< 0.01$. All data are presented as Mean ± SEM. (Statistic methods: two-tailed *t*-test)

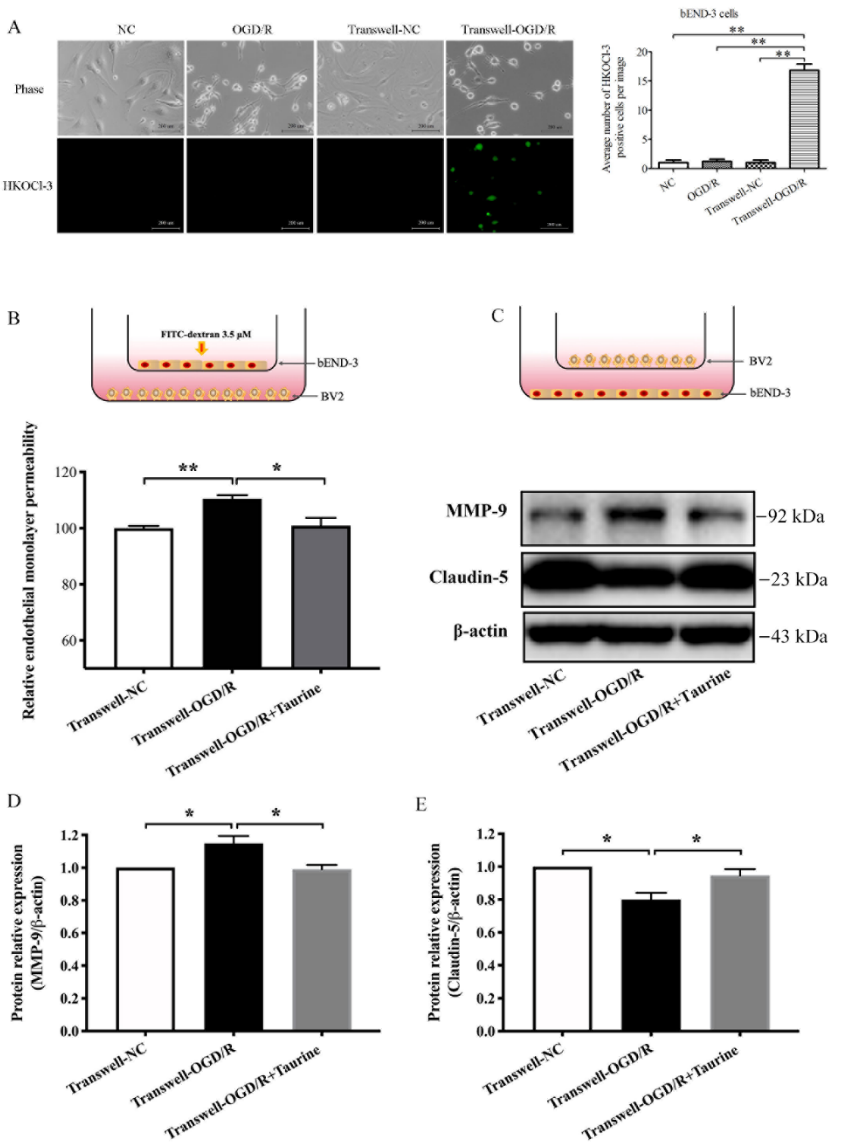

Figure S5: Effect of HOCl on endothelial permeability and expression of MMP-9 and claudin-5 in bEND-3 cells when co-cultured with BV2 under 4 h OGD plus 24 h reoxygenation condition in vitro. In the taurine treatment group, the normal medium with taurine (2 mM) was added at the onset of reoxygenation. (A) HKOCl-3 staining HOCl production from PC12 cells in single cultured plates or co-cultured transwells with BV2 cells under NC or OGD/R condition, bar = 200 μm. (B) In the upper chamber, bEND3 cells were grown on the insert with assay medium containing FITC-dextran. In the lower chamber, BV2 cells were cultured at the plate and endothelial permeability was assessed by measuring the clearance of FITC-dextran from the upper chamber to the lower chamber. (C) In the upper chamber, BV2 cells were cultured in the insert; In the lower chamber, bEND3 cells were cultured at the plate. Western blot analysis detected the expression of MMP-9 and claudin-5 in bEND-3 cells co-cultured with BV2 cells in a transwell co-culture system. (D, E) Quantitative analysis of MMP-9 and claudin-5 expression; N=3. Transwell-OGD/R: BV2 and PC12 co-cultured cells were exposed to 4 h OGD with 24 h of reoxygenation. *$p < 0.05$, **$p < 0.01$. All data are presented as Mean ± SEM. (Statistic methods: one-way ANOVA followed by Tukey's multiple comparisons test).

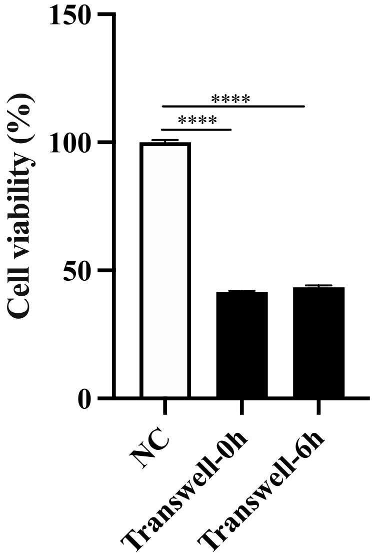

Figure S6: MTT assay for cell viability in BV2 cells co-cultured with PC12 cells under NC or OGD/R conditions after 4 h of OGD and 0 h or 6 h of reoxygenation. Transwell-NC: co-culture BV2 and PC12 cells under normal culture condition; Transwell-0h, 6h: BV2 and PC12 co-culture cells were exposed to 4 h OGD with 0 h or 6 h of reoxygenation. ****$p < 0.0001$. All data are presented as Mean ± SEM. (Statistic methods: one-way ANOVA followed by Tukey's multiple comparisons test).

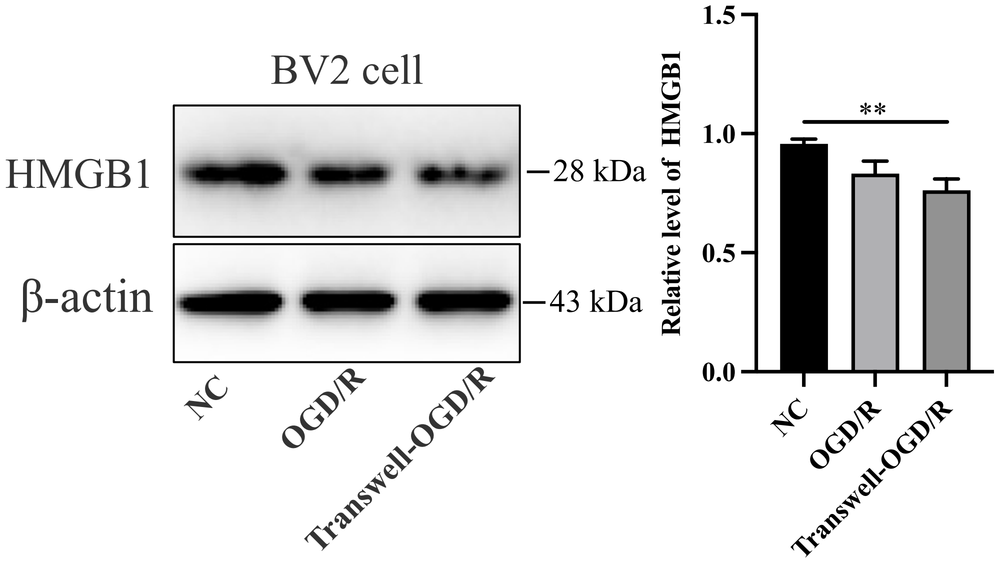

Figure S7: Western blot analysis for HMGB1 expression in BV2 cells under monoculture or co-culture with PC12 cells conditions after 4 h of OGD and 24 h of reoxygenation. Transwell-OGD/R: BV2 and PC12 co-cultured cells were exposed to 4h OGD with 24h of reoxygenation. **$p < 0.01$. All data are presented as Mean ± SEM. (Statistic methods: one-way ANOVA followed by Tukey's multiple comparisons test)
